# The Hidden Crisis in the Times of COVID-19: Critical Shortages of Medical Laboratory Professionals in Clinical Microbiology

**DOI:** 10.1128/jcm.00241-22

**Published:** 2022-06-06

**Authors:** Amy L. Leber, Ellena Peterson, Jennifer Dien Bard

**Affiliations:** a Department of Pathology and Laboratory Medicine, Nationwide Children’s Hospital, Columbus, Ohio, USA; b Ohio State University College of Medicine, Columbus, Ohio, USA; c Department of Pathology and Laboratory Medicine, University of California Irvine, Irvine, California, USA; d Department of Pathology and Laboratory Medicine, Children’s Hospital Los Angeles, Los Angeles, California, USA; e Keck School of Medicine, University of Southern California, Los Angeles, California, USA; Vanderbilt University Medical Center

**Keywords:** microbiology laboratory, workforce shortage, staffing, COVID pandemic, COVID

## Abstract

The COVID pandemic has put a spotlight on laboratory medicine, showcasing how vital diagnostic testing is for society and the health care system. It has also brought to light and accelerated the critical shortage of trained and experienced laboratory personnel that has been felt for decades. The need for laboratory professionals is expected to grow by 11% between 2020 and 2030, a higher rate of growth than the overall average for all other health care occupations. Here, the background to this workforce shortage is reviewed. Some proposed actions to help address the issue are put forth, including increasing awareness of the medical laboratory science profession along with bolstering training opportunities and awareness of alternate routes to obtaining certification as a medical laboratory scientist. In addition, recent survey data specifically related to the employee shortages in microbiology are presented which demonstrate that 80% of microbiology laboratories have vacant positions and that filling these positions is challenging for a number of reasons, including a lack of qualified applicants.

## INTRODUCTION

*There is a saying in the field of laboratory medicine that “the lab” is always hidden in the basement of the hospital.*

There may be some literal truth to this statement. However, metaphorically, the role of clinical laboratories as a vital component of medicine is often “hidden” or under-recognized. The irony is that laboratory medicine is integral to patient care and more than 70% of all patient diagnoses are made because of a laboratory test (1). The COVID pandemic has put a spotlight on laboratory medicine, showcasing how vital diagnostic tests are. To date, hundreds of millions of SARS-CoV-2 tests have been performed in the United States; this is not inclusive of all other diagnostic testing conducting daily in laboratories across the county. As evident throughout the pandemic, particularly with every COVID surge, testing is essential not only for diagnosis of the disease but for supporting public health efforts to curb further spread. Nonetheless, there remains a lack of awareness that with *every single* laboratory test reported, there are clinical laboratorians who were responsible for performing the test and analyzing and reporting the results. As is the case for other medical specialties, the pandemic brought to light and accelerated the critical shortage of trained and experienced laboratory personnel that has been felt for decades. To be clear, diagnostic testing *cannot* exist without qualified medical laboratory professionals. Here, we assess some of the most important factors related to the shortage and describe some actions that can be taken to mitigate the crisis, with a focus on the microbiology laboratory.

It is important that key stakeholders, including hospital administrators, human resource personal within the hospital, and even the wider public are cognizant of the issues so that thoughtful and effective strategies are developed to address the root causes. Clearly, this dire situation will continue to worsen with inaction.

## WHAT’S IN A NAME?

The “lab” is made up of medical laboratory professionals, including both credentialed and non- credentialed personnel. These roles have been given many different titles, including medical technologist, laboratory scientist, and laboratory assistants, which are considered outdated (https://www.ascp.org/content/docs/default-source/boc-pdfs/about_boc/standardizing-the-professional-title-of-medical-laboratory-professionals.pdf, accessed April 4, 2022). The more universally accepted terms are medical laboratory scientist (MLS) and medical laboratory technicians (MLT). An MLS requires a 4-year Bachelor of Science Degree, and an MLT requires a 2-year associate’s degree. Both can pursue credentialing through the American Society for Clinical Pathology (ASCP) or other agencies. The duties associated with the two positions may be different as described by the ASCP, and there are multiple routes to attain certification by ASCP or other organizations. (Table 1). Personnel are trained in different specialties, including microbiology, clinical chemistry, hematology, transfusion medicine, and histology. The extensive training and experience needed to work as an MLS or MLT, in addition to the 4-year or 2-year degrees required, respectively, should not be underestimated. Medical laboratory professionals are required to perform high complexity assays, operate complex instrumentation, perform quality control, interpret results, troubleshoot problems, and provide guidance to those ordering testing as to the appropriateness of the sample type and the individual tests ordered.

**TABLE 1:** Summary of requirements to sit for the ASCP Certification Examination for potential microbiology laboratorians^*a*^

Characteristic	MLT(ASCP)	MLS(ASCP)	Technologist in microbiology [M(ASCP)]
Work duties per ASCP	Collecting and processing biological specimens to perform routine laboratory test in blood banking, chemistry, hematology, immunology, microbiology, molecular biology and/or urinalysis	Conducting a full range of laboratory tests, from routine to complex, in blood banking, chemistry, hematology, immunology, microbiology, molecular biology and/or urinalysis to provide information necessary for the diagnosis and treatment of disease	Identifies bacteria and microorganisms in tissue samples and body fluids to aid in the diagnosis and treatment of infectious diseases
Avg salary^*b*^ ($/hr)	25.75	30.02	NA^*b*^
ASCP certification route			
1	Associate degree or 60 semester hours academic credit AND successful completion of an accredited MLT program within the last 5 yrs	Bachelor’s degree AND successful completion of an accredited MLS program within the last 5 yrs	Valid MT/MLS certification AND bachelor’s degree
2	Valid CLA(ASCP) certification AND associate degree or 60 semester hrs academic credit with 6 hrs in chemistry and 6 in biology	Valid MLT(ASCP) certification AND bachelor’s degree with 16 semester hrs in biology, including 1 semester in microbiology and 16 hrs in chemistry, including 1 semester in organic or biochemistry AND 2 yrs of full-time, acceptable clinical experience within the last 5 yrs	Bachelor’s degree with a major in biological science or chemistry OR bachelor’s degree with a combination of 30 semester hrs in biology and chemistry AND 1 yr of full-time, acceptable clinical experience in microbiology within the last 5 yrs
3	Associate degree or 60 semester hrs of academic credit with 6 semester hrs in chemistry and 6 semester hrs in biology AND successful completion of a 50-wk US military medical laboratory training course within the last 10 yrs	Valid CLA(ASCP) certification AND bachelor’s degree with 16 semester hrs in biology, including 1 semester in microbiology and 16 hrs in chemistry, including 1 semester in organic or biochemistry AND 4 yrs of full-time, acceptable clinical experience in blood banking, chemistry, hematology, microbiology, immunology, and urinalysis/body fluids in an acceptable lab within the last 8 yrs	Bachelor’s degree with a major in biological science or chemistry OR a bachelor’s degree with a combination of 30 semester hrs in biology and chemistry AND successful completion of a structured program in microbiology under the auspices of a NAACLS-accredited MLS program within the last 5 yrs
4	Associate degree or 60 semester hours with 6 semester hrs in chemistry and 6 hrs in biology AND 3 yrs of full-time, acceptable clinical experience in blood banking, chemistry, hematology, microbiology, immunology, and urinalysis/body in an acceptable lab within the last 3 yrs	Bachelor’s degree with 16 semester hours in biology, including one semester in microbiology and 16 semester hrs in chemistry, including one semester in organic or biochemistry AND 5 yrs of full-time, acceptable clinical experience in blood banking, chemistry, hematology, microbiology, immunology, and urinalysis/body fluids in an acceptable lab within the last 10 yrs	Master’s degree in microbiology or a related field AND 6 mo of full-time, acceptable clinical experience in microbiology in an acceptable lab within the last 5 yrs
5	NA	Valid MT/MLS certification AND a transcript evaluation verifying equivalency to a US bachelor’s degree AND 5 yrs of full-time, acceptable clinical experience in blood banking, chemistry, hematology, microbiology, immunology, and urinalysis/body fluids in an acceptable lab within the last 10 yrs	Bachelor’s degree in medical laboratory science OR a bachelor’s degree with a combination of 30 semester hrs in biology and chemistry AND successful completion of an NAACLS-accredited MLS program within the last 5 yrs

a MLT, medical laboratory technician; MLS, medical laboratory scientist; NA, not applicable; CLA, certified laboratory assistant; NAACLS, National Accrediting Agency for Clinical Laboratory Sciences. This table is not meant to account for all the rules and requirements for ASCP certification. For full details, see https://www.ascp.org/content/docs/default-source/boc-pdfs/exam-content-outlines/ascp-boc-us-procedures-book-web.pdf.

b Source: Garcia et al. (2).

It is important to note that the Clinical Laboratory Improvements Amendments (CLIA) only require that those performing moderate and high complexity testing have an associate’s or bachelor’s degree with specified course work in various sciences (https://documents.cap.org/documents/CAP-personnel-requirements-by-test-complexity.pdf, accessed April 4, 2022). No certification or licensure is required. However, hiring individuals with no specific skills or experience in laboratory science is a heavy burden for employers in terms of training, productivity, and quality. Therefore, employers often seek individuals with certification by an appropriate body (i.e., the ASCP). In addition, depending on the state, licensure may also be required (i.e., in California). One consequence of hiring noncertified or nonlicensed individuals is that the salary expectations are often lower, which unfortunately shifts the overall average salary documented for the profession. Therefore, recognition of the importance of appropriate training programs, certification, and licensure is essential for the field of laboratory medicine to grow and for practitioners to be recognized as highly trained and qualified members of the health care team.

## THE LABORATORY CRISIS

To understand the current crisis, both historical and current data are informative. According to the US Bureau of Labor Statistics (BLS), which provides data and forecasting on hundreds of occupations yearly (https://www.bls.gov/ooh/healthcare/clinical-laboratory-technologists-and-technicians.htm, accessed April 4, 2022), there were 335,500 individuals employed as “clinical laboratory technologists” and “clinical laboratory technicians” in 2020 combined. Of note is the use of outdated terms for medical laboratory scientist and medical laboratory technicians. In addition, while convenient for the purpose of obtaining a number, the grouping of these two job categories into one is riddled with inaccuracies because these two positions have different educational and training requirements and distinct salary ranges. Merging the two separate professions into one validates the concerns raised regarding the lack of appreciation and understanding for these professions within laboratory medicine. This would be akin to combining the numbers for registered nurses and licensed practical nurses. Nonetheless, these are the only comparative data currently available from a single source. The average yearly wage listed in the BLS database for this combined category is $54,180. A more refined data set from the ASCP 2019 Wage Survey found that the average hourly rate for staff-level MLS was $30.02 ($62,441.60 per year) versus $25.75 ($48,235.20 per year) for MLT (2). However, the salary range can differ greatly depending on the city or state of employment for an MLS or MLT. In 2019, the highest average hourly wage for an MLS was $52.71 in California while the lowest was $25.74 in South Dakota (2). For MLT, the highest average hourly wage was $35.73 in California and the lowest was $18.64 in Mississippi.

The need for laboratory professionals is expected to increase by 11% between 2020 and 2030, a faster growth rate than the overall average for all other health care occupations (https://www.bls.gov/ooh/healthcare/clinical-laboratory-technologists-and-technicians.htm, accessed April 4, 2022). Data generated from a 2018 survey by the ASCP report that the average staff vacancy rate for the clinical laboratory was 9.1% overall and 10.6% in microbiology specifically (3). The next ASCP vacancy survey, in 2020, showed an overall improvement, with average staff vacancy rates in clinical laboratory of 8.5% overall and 6.9% in microbiology specifically (4). However, considering those leaving the workforce, this increase is insufficient: the rates of anticipated staff-level retirements in the next 5 years were 14.5% overall, 12.8% in microbiology, and 30.9% for microbiology supervisors. (4). Overall staffing shortages were reported to be highest in the Central Northeast (10.2%; Illinois, Indiana, Michigan, Ohio, and Wisconsin), whereas the lowest vacancy rates were found in the Central Southwest (5.3%; Arkansas, Louisiana, Oklahoma, and Texas). One noteworthy finding is that while shortages were an issue across the entire health care spectrum, they were particularly challenging in rural areas (4). The impact on staffing early in the COVID pandemic was accounted for in the 2020 ASCP Vacancy Survey, as the data were collected in June and July of 2020. However, the situation is complex, because during the survey period, many laboratories were experiencing lower workloads due to shutdowns caused by the pandemic (4). More data are needed to understand the ultimate impact of the pandemic on laboratory staffing and related issues such as early retirement and the lack of graduating MLS and MLT students.

A comprehensive analysis of the clinical laboratory workforce challenges was recently published by the ASCP and the Center for Health Workforce Studies at the University of Washington (5). The findings of this study emphasized the need for action to ensure that future workforce needs are met. The overriding goals of this study were improving the visibility of clinical laboratory occupations, including a better understanding of what this career entails; improving workforce recruitment and retention; and focusing on diversity and inclusion in the laboratory. One of the major problems affecting vacancy rates is that there are fewer graduates than vacancies for medical laboratory professionals. This is partially due to fewer training programs. In the United States., there are college-level programs for both MLS, a 4-year Bachelor of Science degree, and MLT, which requires a 2-year associate’s degree. According to the National Accrediting Agency for Clinical Laboratory Sciences (NAACLS), the number of MLS programs declined by 57.2%, from 638 to 273, in the 16-year period between 1983 to 1999, with enrollment declining from 8,296 to 5,117 during that time (http://www.captodayonline.com/Archives/feature_stories/feat2900.htm, accessed April 4, 2022). In 2021, there were 240 MLS and 238 MLT training programs accredited by the NAACLS, with 4,114 and 2,844 graduates, respectively (NAACLS, personal communication). While these numbers have been relatively steady for the past 10 years, they are far from sufficient to meet current and future needs.

## EXPLORATION OF THE CRISIS WITHIN ONE SPECIALTY

**FIG 1:**
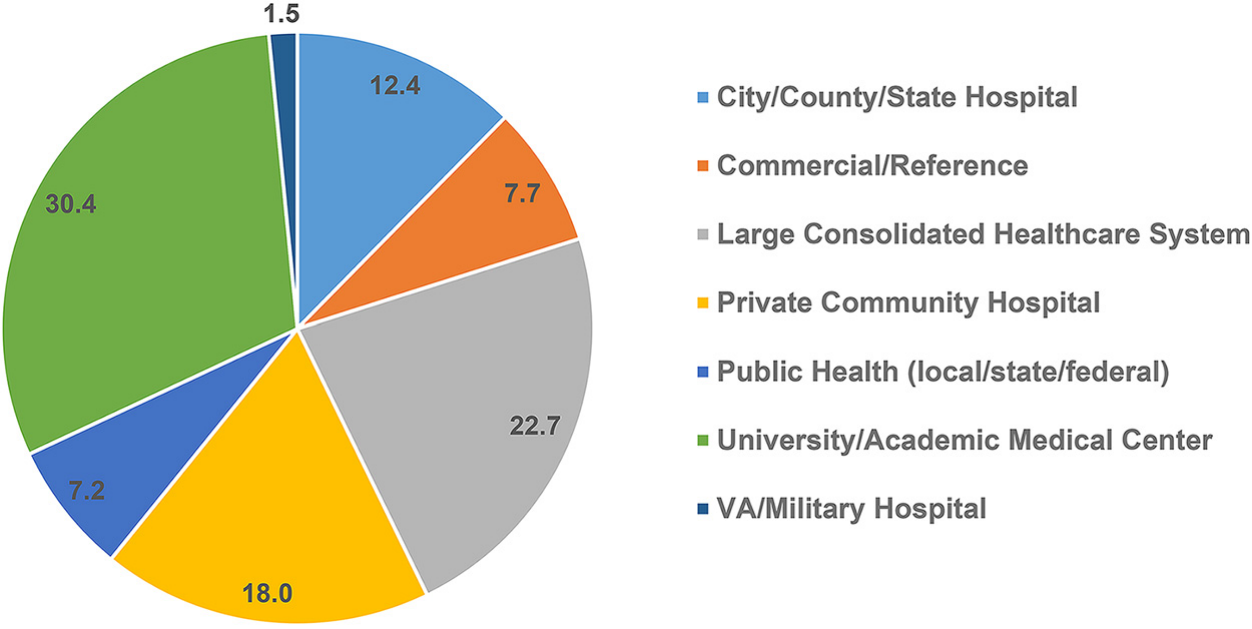
Laboratory type of survey respondents (%, *n* = 194).

Because the American Society for Microbiology (ASM) is a professional organization that supports clinical microbiologists as one of its constituent groups, it seems appropriate for this organization to be proactive in addressing this critical problem. The Personnel Standards and Workforce Committee (PSW) is a subcommittee within ASM devoted to understanding the issues and standards associated with the clinical microbiology workforce. In 2021, the committee set about to collect contemporary data from ASM members, specifically those involved in the clinical microbiology laboratory. The PSW produced and distributed a survey, entitled the “ASM Clinical Microbiology Workforce Survey”, on two ASM-sponsored list serves (ClinMicroNet and DivCNet). The survey was conducted from May 3, 2021 to June 21, 2021 and included a total of 18 questions geared toward understanding laboratory demographics, vacancy rates, and other challenges. Respondents represented 194 unique clinical or public health microbiology laboratories from a wide range of laboratory settings. A portion of the data is presented here. Participants came from 44 states plus the District of Columbia. See Tables 2 and 3 and Fig. 1 for details of the survey and respondents. The full survey is available in the supplementary material.

**TABLE 2:** Microbiology testing volumes of survey respondents

Yearly testing vol	*n*	%
<5,000	5	2.6
5,000−49,999	27	13.9
50,000−99,999	28	14.4
100,000−199,999	31	16.0
200,000−499,999	51	26.3
500,000−999,999	34	17.5
>1,000,000	14	7.2
Unknown	4	2.1

**TABLE 3:** Breakdown of MLS employed in microbiology laboratories^*a*^

Lab type	MLS, *n* (% of lab type)				
	<10	10−24	25−49	50−75	>75
City/county/state hospital (*n* = 24)	20 (83.3)	3 (12.5)	1 (4.2)	0 (0)	0 (0)
Commercial/reference (*n* = 15)	2 (13.3)	5 (33.3)	3 (20.0)	1 (6.7)	4 (26.7)
Large, consolidated healthcare system (*n* = 44)	10 (22.7)	9 (20.5)	20 (45.5)	4 (9.1)	1 (2.3)
Private community hospital (*n* = 35)	21 (60.0)	10 (28.6)	4 (11.4)	0 (0)	0 (0)
Public health (*n* = 14)	3 (21.4)	4 (28.6)	6 (42.9)	1 (7.1)	0 (0)
University/academic medical center (*n* = 59)	5 (8.5)	20 (33.9)	29 (49.2)	4 (6.8)	1 (1.7)
VA/military hospital (*n* = 3)	1 (33.3)	2 (67.7)	0 (0)	0 (0)	0 (0)

a MLS, medical laboratory scientist.

### There is indeed a workforce shortage in clinical microbiology.

**FIG 2:**
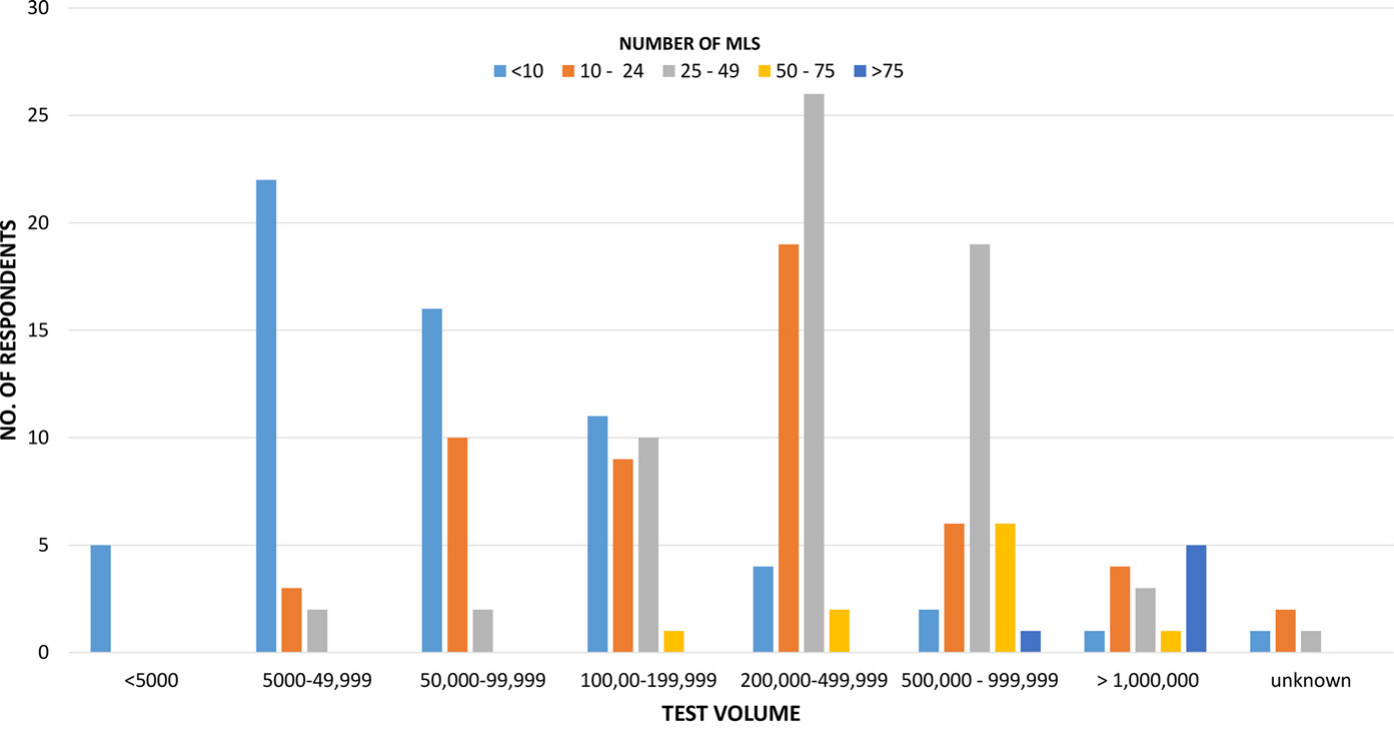
Breakdown of staffing numbers for medical laboratory scientists compared to annual test volume in microbiology laboratories (*n* = 194).

The ASM Workforce Survey showed that the number of MLS personnel employed varied greatly across institutions, with more than 50% of county/state hospital and community hospital laboratories employing less than 10 MLSs in the microbiology laboratory, whereas laboratories with more than 50 MLS were more likely to be reference, health system, and academic medical center laboratories (Table 3). The number of MLSs compared to testing volumes is presented in Fig. 2. As expected, we observed a strong correlation between testing volumes and staffing numbers, with some exceptions.

**FIG 3:**
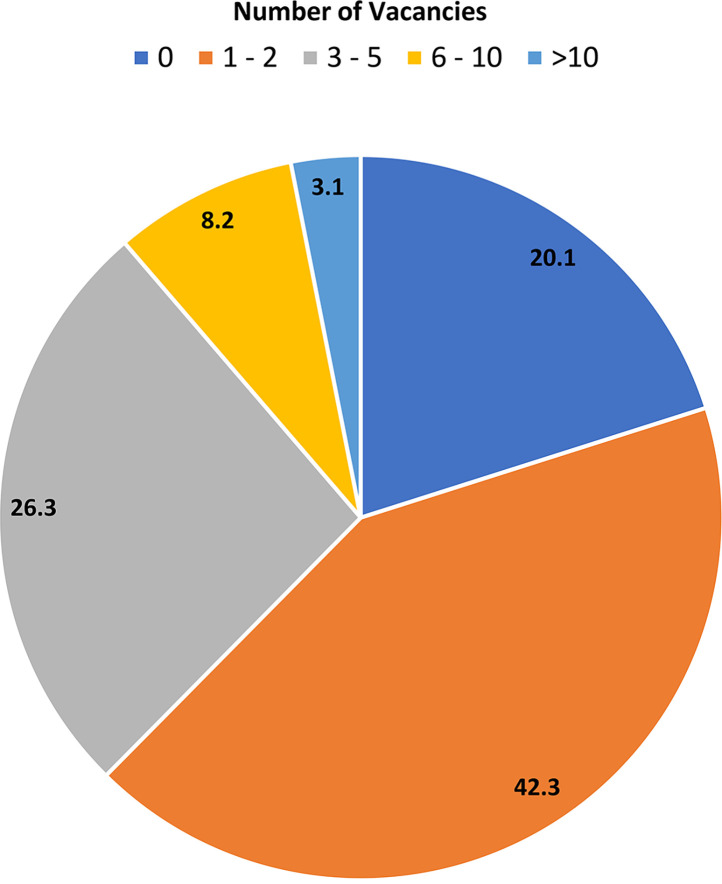
Number of vacancies for medical laboratory scientists in the microbiology laboratory (%, *n* = 194).

The ASM Workforce Survey confirmed that clinical microbiology laboratories continue to experience significant shortages of qualified personnel. Of the 194 participants, over 80% reported at least one open position for MLS, with over one-third having between 3 and 5 vacant positions. Six (3.3%) laboratories had more than 10 vacancies each (Fig. 3). The level of vacancies for MLTs was lower, with 53% reporting one or more vacancies (see question 12 in the supplemental material). Vacancy woes were evident across all institutions, including large reference laboratories, academic medical centers, and private community hospitals. (Table 4).

**TABLE 4:** Distribution of vacancies for MLS in clinical microbiology laboratories^*a*^

Lab type	MLS vacancies, *n* (% of lab group)				
	0	1−2	3−5	6−10	>10
City/county/state hospital (*n* = 24)	7 (29.2)	16 (66.7)	1 (4.2)	0 (0)	0 (0)
Commercial/reference (*n* = 15)	3 (20.0)	5 (33.3)	3 (20.0)	2 (13.3)	2 (13.3)
Large, consolidated healthcare system (*n* = 44)	7 (15.9)	16 (36.4)	16 (36.4)	4 (9.1)	1 (2.3)
Private community hospital (*n* = 35)	8 (22.9)	19 (54.3)	7 (20.0)	1 (2.9)	0 (0)
Public health (*n* = 14)	3 (21.4)	5 (35.7)	5 (35.7)	0 (0)	1 (7.1)
University/academic medical center (*n* = 59)	11 (18.6)	18 (30.5)	19 (32.2)	9 (15.3)	2 (3.4)
VA/military hospital (*n* = 3)	0 (0)	3 (100)	0 (0)	0 (0)	0 (0)

a MLS, medical laboratory scientist.

### Significant delays and hurdles to filling vacancies.

The amount of time required to fill MLS vacancies differed across institutions but notably, 66% of survey respondents estimated at least a 4-month time frame to fill a vacant position (Table 5). Moreover, 73% of respondents indicated that once vacated, positions needed to be re-approved at the administrative level prior to being posted (see question 16 in the supplemental material).

**TABLE 5:** Time to fill vacant medical laboratory scientist positions in microbiology

Time	*n*	%
<1 mo	5	2.6
1–3 mo	61	31.4
4–6 mo	83	42.8
>6 mo	45	23.2

**FIG 4:**
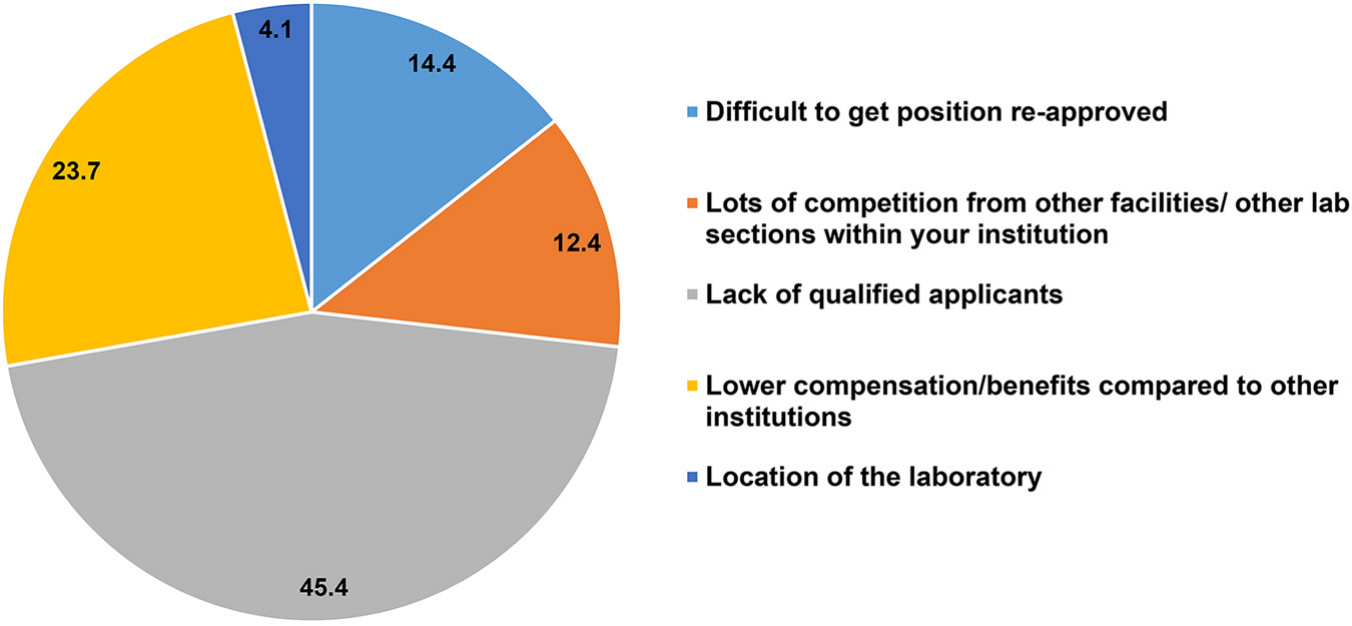
Biggest obstacles to filling vacant medical laboratory scientist positions in microbiology. (%, *n* = 194).

When asked to rank the most challenging issue related to filling vacancies in the clinical microbiology laboratory, 45% of the respondents ranked the lack of qualified applicants as the biggest obstacle. The other challenges were, in order of importance, lower compensation compared to other institutions, difficulty getting positions (re)-approved, competition from other facilities or lab sections within the same institution, and laboratory location (Fig. 4). The shortage of available, qualified personnel has escalated competition, within institutions in the same region vying for the limited pool of applicants. This often leads to low retention in smaller institutions which may not have the same pull compared to larger players in the field. This low retention contributes to the problem due to difficulties in getting the *same* positions re-approved and was indicated by 15% of respondents as the top obstacle.

Similar challenges were echoed in the recent ASCP and University of Washington study. Data from focus groups showed that the level of compensation and competitive hiring for MLSs and MLTs often lead to vacant positions, especially in rural areas (5). The aging workforce and impact of retirement also contributed to the situation for both MLTs and MLSs.

### Training microbiologists doesn’t happen overnight.

**FIG 5:**
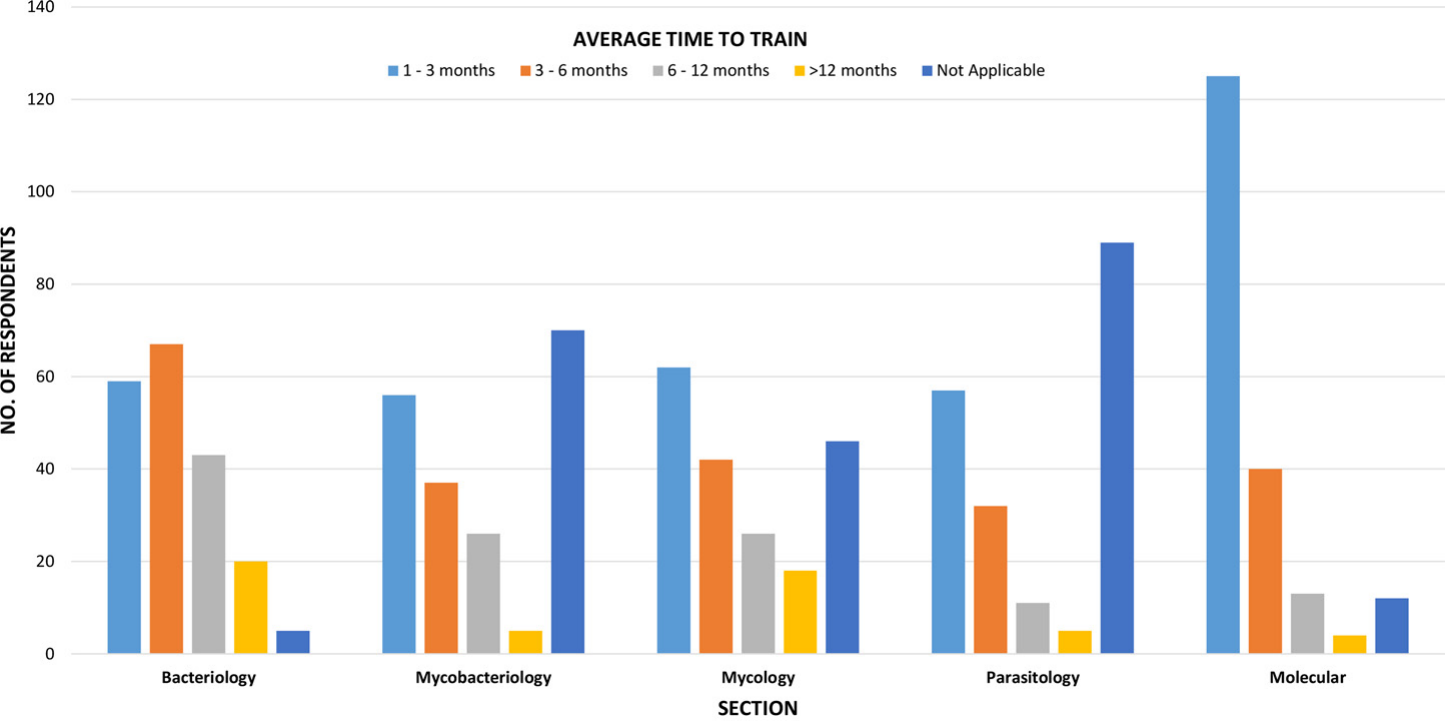
Average amount of time required to train a new medical laboratory scientist in microbiology sections (*n* = 194).

Clinical microbiology is a highly specialized section of laboratory medicine and one of the few specialties that remain very manual, with interpretations being highly subjective and dependent on knowledge and expertise. New graduates from an MLT or MLS program still require a significant amount of time to train. In the ASM Clinical Microbiology Workforce Survey, participants were asked about training times for the different sections. As seen in Fig. 5, it can often take at least 6 months to complete initial training in a given area. Depending on the laboratory section, proficiency may not be reached for at least 1 year. Bacteriology, parasitology, and mycology are examples of sections where expertise in morphology and phenotypic characteristics is imperative for accurate diagnostic workup; however, such expertise requires training and experience. Moreover, the MLSs who possess the expertise unfortunately often retire *before* all of their knowledge can be passed on to the new generation.

## CONCERNS FOR QUALITY AND PATIENT SAFETY

With the mounting pressure of increasing workloads, short staffing, and a lack of experienced laboratory personnel, a worrying picture is emerging. This has been particularly evident during the COVID pandemic, and it is imperative for hospital administrators to not discount these issues. Our ability to provide reliable and timely laboratory results and ensure patient safety is in jeopardy. In the National Academy of Science publication “To Err is Human: Building a Safer Heath System,” the conditions which create medical errors are described as multifactorial (6). Many are related to the complexity of the systems in which we work; however, human error is one of the greatest contributors. To maintain safety, we need a well-trained and knowledgeable workforce, adequate staffing levels with reasonable work schedules, and systems that support efficient and safe work environments. A prime example that highlights this issue is Gram stain interpretation. While experienced microbiologists take this skill for granted, the importance of interpreting a Gram stain is even more profound in the absence of skilled and experienced MLSs. While correct reporting of a Gram stain can allow for appropriate modification of antimicrobial therapy, the misinterpretation of a Gram stain can be detrimental (7).

When staffing and training falter, the risk of errors increases. The breadth of diagnostic services offered can be negatively impacted. Constraints endured during the COVID pandemic led to laboratories restricting or discontinuing clinical tests and sending testing to reference laboratories. However, these are not long-term solutions, as reference laboratories are also clinical laboratories which need the same skilled MLSs and MLTs. We must address the issues which impact the lack of a robust and well-trained laboratory workforce.

## WORKING TOWARD SOLUTIONS

The root causes for the shortages are multiple, and solutions will require creativity and commitment (5, 8). It is abundantly clear, especially in light of the current pandemic, that these shortages mean that the clinical laboratory testing system in the US is vulnerable and reaching crisis levels. Perhaps wider recognition of this problem will bring momentum for fundamental changes at the hospital administrator level.

Many groups have been working to determine the causes and implement solutions. Some key areas of focus to move the needle on this issue include the following:

- **Investment in training programs.** While colleges and universities are facing many challenges, including decreased funding, it is necessary to invest in MLS and MLT training. It also seems prudent to call on those in health care leadership roles to advocate for increasing training programs at the post-secondary level and exposing students to the possibility of a career in laboratory medicine where possible. In addition, state and federal governments should invest in education of laboratory professionals similar to what has been done for nursing. As part of H.R. 1865, $259,972,000 was provided for Title VIII Nursing Workforce Development Programs in fiscal year 2020, which funds nursing education and research (https://www.congress.gov/bill/116th-congress/house-bill/1865/text, accessed April 4, 2022).
- **Engage clinical rotation sites.** One of the biggest obstacles to existing training programs is the limited number of laboratories that are willing to serve as sites for the practicum period. This in-laboratory, hands-on training is part of the MLS and MLT degree programs. A push at the laboratory administration level to approve training sites for MLS or MLT students is needed. Although this does require some investment, the benefit is exponential, as it allows institutions the ability to retain trainees as permanent employees. ASM has begun to engage with clinical laboratories to identify those that can host students for clinical microbiology rotations. ASM formed a partnership with Weber State University (WSU) to offer a microbiology certificate program. WSU offers students didactic content, and ASM member laboratories offer a minimum of four weeks of training, specifically in microbiology. Students complete their coursework online and select a conveniently located training site. The program targets students and professionals (note: this program is only available to students in the US and is not recognized in CA or NY) with a bachelor’s degree in a qualifying field (e.g., biological science or chemistry) and prepares them to be eligible for the Technologist in Microbiology M(ASCP) certification examination from ASCP via Route 3 (Table 1). Since the program’s launch in September 2021, 20 laboratories have agreed to serve as training sites, and 12 students are enrolled at the time of writing. A collaboration with other societies such as the ASCP and the American Association of Clinical Chemistry (AACC) to create a clearing house which identifies laboratory training opportunities and shares that information with prospective trainees would be useful not only for microbiology but for other areas of the clinical laboratory, such as chemistry and hematology.
- **Expand innovative and alternative career routes.** Traditional brick and mortar, in-person education may need to be replaced or supplemented with online learning curricula that offer accredited programs in medical laboratory science. Currently, the number of these programs is small but growing (https://www.medicaltechnologyschools.com/medical-lab-scientist/online-mls-degree-programs, accessed April 4, 2022). Additional programs would accelerate the number of trained graduates. Here, clinical training sites will still need to be expanded. As mentioned above, for individuals with a four-year bachelor’s degree, successful completion of a structured program in microbiology under the auspices of a NAACLS-accredited MLS program can qualify them for credentialing as a Technologist in Microbiology [M(ASCP)] via Route 3. Similar credentialing can be obtained for technologists in blood banking, chemistry, cytogenetics, cytotechnology, hematology, and molecular biology. Also, for those with a bachelor’s degree in science, there is a route to MLS certification that requires one year of on-the-job training. ASCP Route 2 requires that the applicant have obtained one year of full-time, acceptable experience in an acceptable clinical laboratory within the past five years. This so-called “4 plus 1” path has specific requirements, but it is a viable alternative to the MLS degree route. Both of these routes may be great options for those graduating with a bachelor’s degree and uncertainty about their career path. See Table 1 for a summary of the routes to certification. Each laboratory should explore whether providing in-laboratory training for these pathways is viable and might provide a way to hire trained, certified, and “home-grown” talent.
- **Increase awareness of the profession for recruitment and retention.** There may be a large untapped pool of potential clinical laboratorians among those who are *unaware* of the existence of the profession, and one can only increase recruitment and training among those who are *cognizant* of this as a career option. There is an important need to work with universities, colleges, hospitals, and other medical institutions to advocate for the medical laboratory profession as a viable and essential part of health care. Starting at the high school level by reaching out to STEM programs, societies such as ASM and ASCP are actively working to help promote the profession. In addition to increasing awareness, attention must be paid to the recruitment and retention of experienced employees. Some obstacles to workforce retention have been identified as limited potential for advancement, high workload, stress, limited availability of training and continuing education, and low salaries.
- **Move toward more equitable compensation.** Compensation levels being lower than those of equivalent medical professionals with similar educational requirements is perhaps one of the key elements in the inability to attract and retain laboratory staff. This should be addressed on both the local and national levels, including providing governmental support. As mentioned above, the median salary for laboratory professionals is lower than for that of some other allied health professionals with similar educational requirements (Table 6). This gap must be recognized and addressed if we hope to change the trajectory of this hidden crisis. Some additional approaches for recruitment and retention strategies have included signing bonuses, referral bonuses, and increasing benefits and perks; however, the effectiveness of these strategies has not been well studied.

**TABLE 6:** Comparison of pay and employment changes for allied health professions^*a*^

Occupation	2020 median pay ($/yr)	Employment change (%), 2020−2030^*b*^
Clinical Laboratory Technologists (MLS) and Technicians (MLT)^*c*^	54,180	11
Registered Nurse	75,330	9
Licensed Practical Nurse	48,820	9
Respiratory Therapist	62,810	23
Radiologic Technician	63,710	9
Dental Hygienist	77,090	11

a MLS, medical laboratory scientist; MLT, medical laboratory technician. Data obtained from US Bureau of Labor Statistics (https://www.bls.gov/ooh/healthcare, accessed April 4, 2022).

b Projected percent change in employment from 2020 to 2030. The average growth rate for all occupations is 8%.

c Job categories not listed separately. ASCP 2019 Wage Survey found that the average hourly rate for staff-level MLS was $30.02 ($62,441.60 per yr) versus $25.75 ($48,235.20 per yr) for MLT (2).

The medical laboratory profession has been and continues to be in a crisis, with too few people in the field to meet needs for trained and qualified personnel. Remaining complacent will be detrimental to this profession. While some are turning to automation as a way to improve efficiency and alleviate the shortage of trained laboratory personnel (9), that approach is unlikely to address the dire staffing needs that many institutions are facing currently and in the years to come. As is the case for our colleagues in nursing, these staffing shortages are affecting our ability to provide consistent, high-quality results for patient care. The nursing shortage may be more tangible, as it is patient-facing. The laboratory, with its proverbial location “in the basement” of the hospital, may lack visibility, leading to an underestimation of our role in the continuum of care. We urge laboratory and hospital administrators, and indeed all vested parties, to recognize the laboratory workforce shortage and look within their institution to identify and address the obstacles which may be present, but also to leverage their positions to advocate for support.
