# Supplementary material for: The Hidden Crisis in the Times of COVID-19: Critical Shortages of Medical Laboratory Professionals in Clinical Microbiology
Source: J Clin Microbiol. 2022 Jun 6;60(8):e00241-22. doi: 10.1128/jcm.00241-22 (PMC9383190; doi:10.1128/jcm.00241-22)
Supplement: Supplemental file 1 — Survey questions 1 to 19 and additional analysis. Download jcm.00241-22-s0001.pdf, PDF file, 1.0 MB [file jcm.00241-22-s0001.pdf]

2021 Clinical Microbiology Workforce Survey

- From the Personnel Standards and Workforce Sub-Committee of the Clinical and Public Health Microbiology Committee, American Society for Microbiology

OVERVIEW:

The COVID pandemic has put a spotlight on laboratory medicine, showcasing how vital diagnostic testing is for the healthcare system and society. It has also brought to light and accelerated the critical shortage of trained and experienced laboratory personnel that has been felt for decades. The need for laboratory professionals is expected to grow by 11% between 2020 and 2030, a rate of growth considered faster than the overall average of all other healthcare occupations. (1) As the American Society for Microbiology is a professional organization that supports clinical microbiologist as one of its constituent groups, it seems appropriate for this organization to be proactive in addressing this critical problem. The Personnel Standards and Workforce Committee (PSW) is a sub-committee within ASM devoted to understanding the issues and standards associated with clinical microbiology workforce. In 2021, the committee set out to collect contemporary data from ASM members, specifically those involved in the clinical microbiology laboratory.

METHODS:

The PSW produced and distributed an ad-hoc survey, entitled the *ASM Clinical Microbiology Workforce Survey*, on two ASM sponsored listservs (ClinMicroNet and DivCNet). The survey was conducted from May 3, 2021 to June 21, 2021 with questions geared towards understanding laboratory demographics, vacancy rates, and other challenges. A total of 210 individuals responded, with 16 being from the same institution as another respondent and were therefore eliminated. This left 194 unique clinical or public health microbiology laboratories from a wide range of laboratory settings. Participants came from 44 states plus the District of Columbia.

Here the PSW committee provides the results of the survey and some additional analysis on the data collected. Our hope is that it will be useful for clinical microbiology laboratories to provide comparative data to support both local and national efforts to improve the shortage of trained personnel. A commentary related to this subject has been published in the *Journal of Clinical Microbiology* (insert reference or link).

If you have any comment or questions related to the survey or the workforce shortage, please contact Peggy McNult (pmcnult@asmusa.org) at ASM.

(1) https://www.bls.gov/ooh/healthcare/clinical-laboratory-technologists-and-technicians.htm, accessed January 18, 2022

(2) ClinMicroNet is composed of an international group of clinical microbiology laboratory directors who openly and daily communicate with one another thorough this medium. The criteria for membership in ClinMicroNet are a Contributing Member of ASM and are Doctoral-level clinical microbiology laboratory director or Laboratory manager with national standing and peer recognition. (3) The goal of the DivCNet is to improve communication among clinical microbiology laboratories and ultimately, improve patient care. This forum may be used to discuss any topic related to clinical microbiology, ASM, ASM Division C, or other topics of interest to the membership.

RESULTS:
The questions are presented along with the results for the survey. These are grouped into four categories: Demographics, Oversite and Management, Staffing and Vacancies, Training, Certification and Challenges

DEMOGRAPHICS

Q1. What position do you hold in the microbiology lab?
Of the respondents, 40.2% indicated a title of Manager/Supervisor and 35.6% indicated a title of Assistant/Associate/Director.

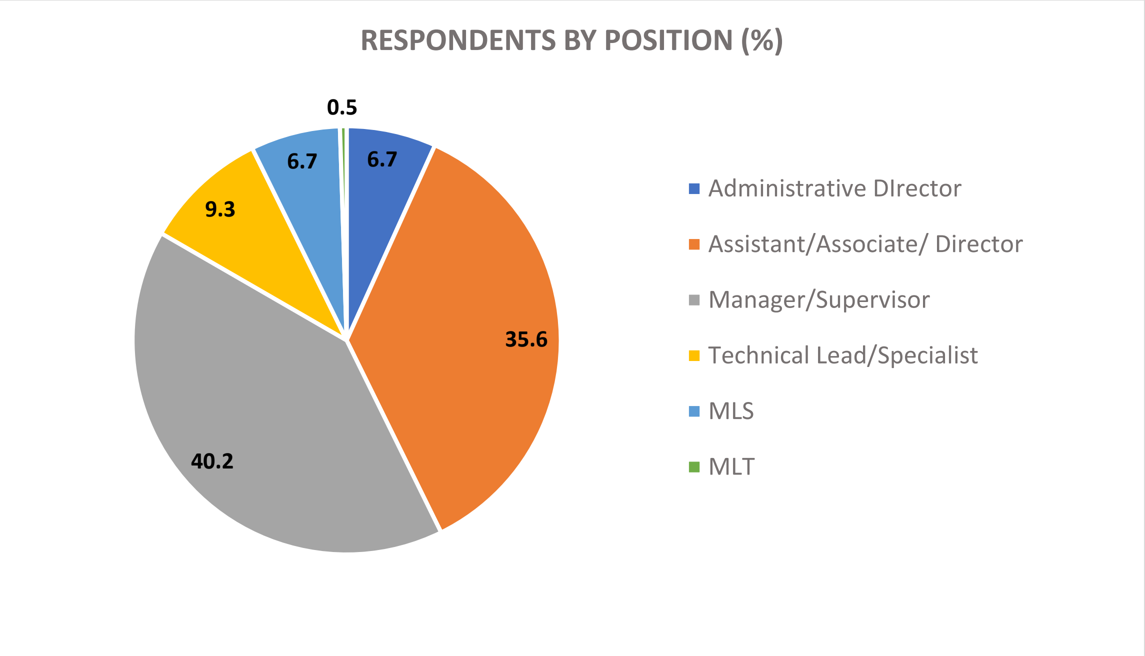

Position	N	%
Administrative Director	13	6.7
Assistant/Associate/ Director	71	36.6
Manager/Supervisor	78	40.2
Technical Lead/Specialist	18	9.3
Medical Laboratory Scientist (MLS)	13	6.7
Medical Laboratory Technician (MLT)	1	0.5

Q2. Which laboratory area(s) do you work in or oversee? Check all that apply.
Of the respondents, 96.9% indicated working in Microbiology followed by Molecular Microbiology (68.6%). Of the 194 respondents, 74.2% responded that they work/oversee more than one area.

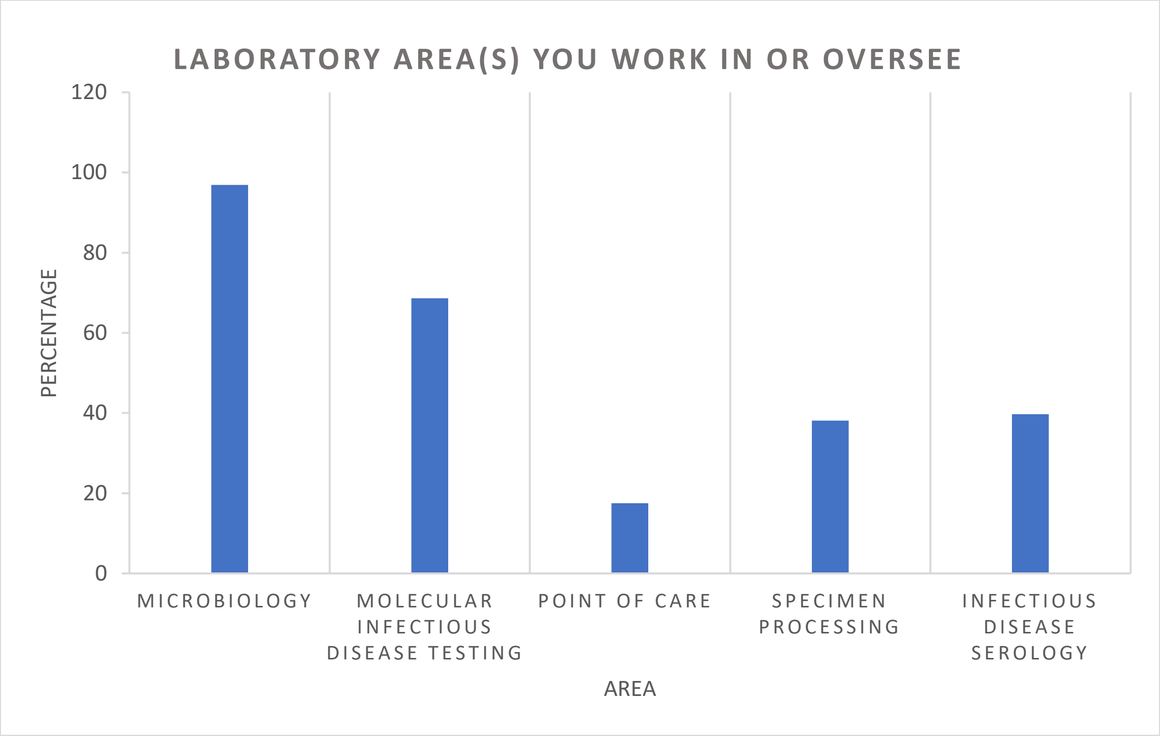

Area	N	%
Microbiology (including sample to answer molecular)	188	96.9
Molecular Infectious Disease Testing (high complexity molecular testing)	133	68.6
Point of Care Testing	34	17.5
Specimen Processing	74	38.1
Infectious Disease Serology	77	39.7

Q3. Which of the following best describes your laboratory?
The largest segment of respondents was from University/Academic Medical Centers. The smallest number of respondents were from Public Health and VA/Military Hospital laboratories.

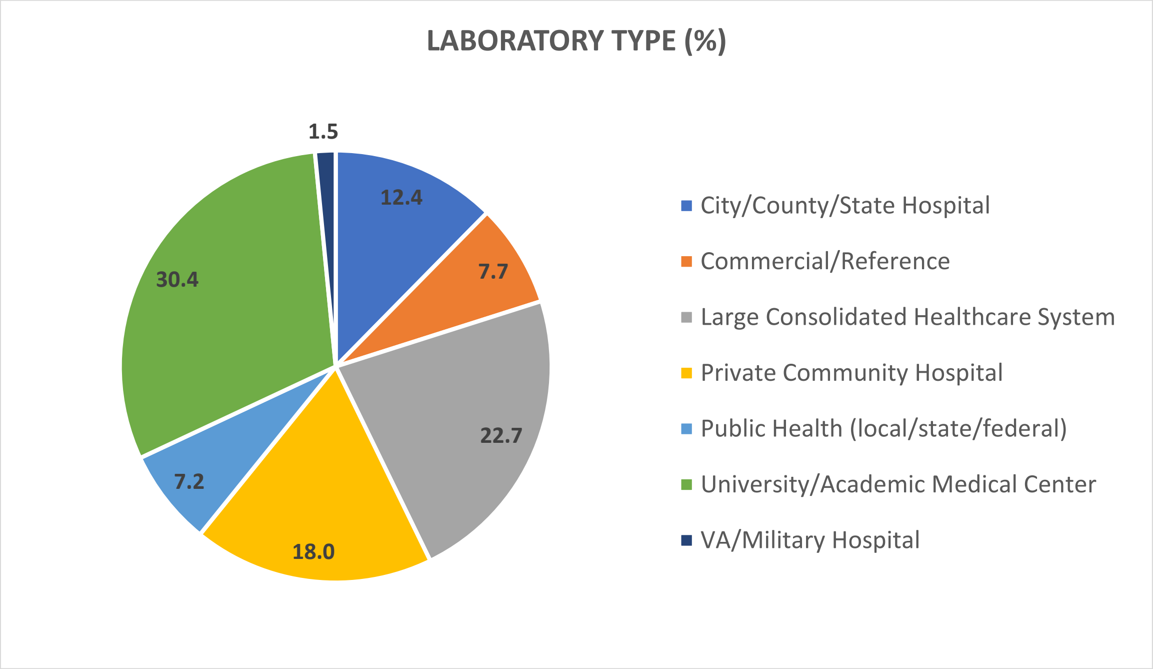

Type of Laboratory	N	%
City/County/State Hospital	24	12.4
Commercial/Reference	15	7.7
Large Consolidated Healthcare System	44	22.7
Private Community Hospital	35	18.0
Public Health (local/state/federal)	14	7.2
University/Academic Medical Center	59	30.4
VA/Military Hospital	3	1.5

Q4. How many diagnostic tests does your microbiology laboratory perform annually?

Of the respondents, approximately 45% reported volumes <200,000 while the remaining 55% reported volumes >200,000.

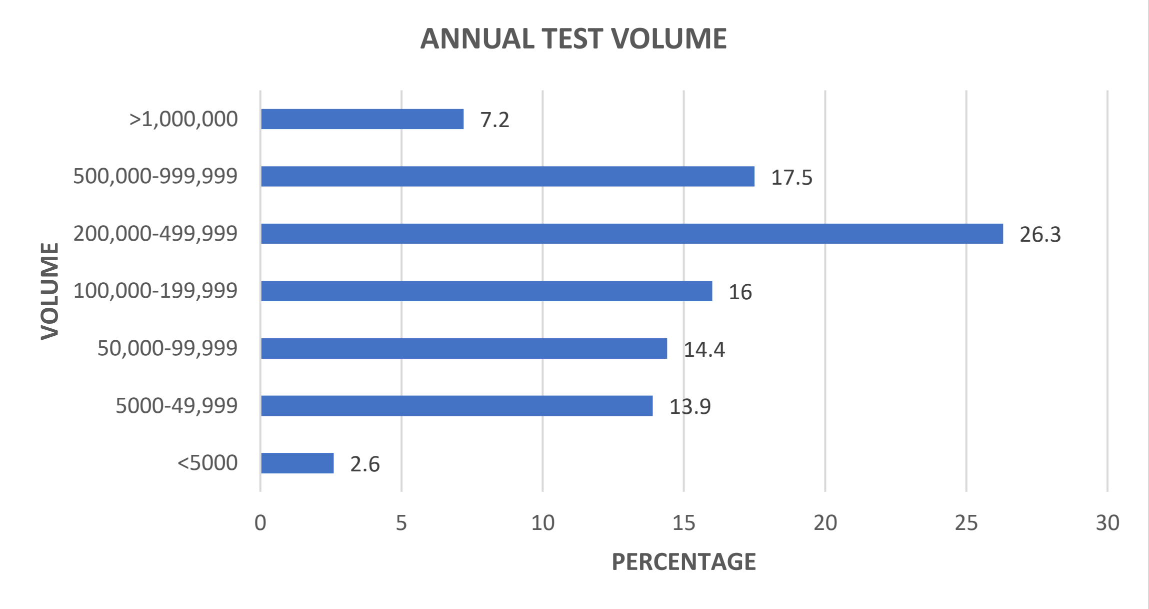

Annual Test Volume	N	%
<5000	5	2.6
5000-49,999	27	13.9
50,000-99,999	28	14.4
100,000-199,999	31	16.0
200,000-499,999	51	26.3
500,000-999,999	34	17.5
>1,000,000	14	7.2
Unknown	4	2.1

Q5. How many shifts does your microbiology laboratory operate? Choose all that apply.
Of the respondents, 89.2% indicated that their respective laboratory operates on first shift. (This may indicate a misinterpretation of the question as it is assumed all labs operate on 1st shift.) The level of labs responding they operated on 2nd shift was 69.6% followed by 59.8% on third shift.

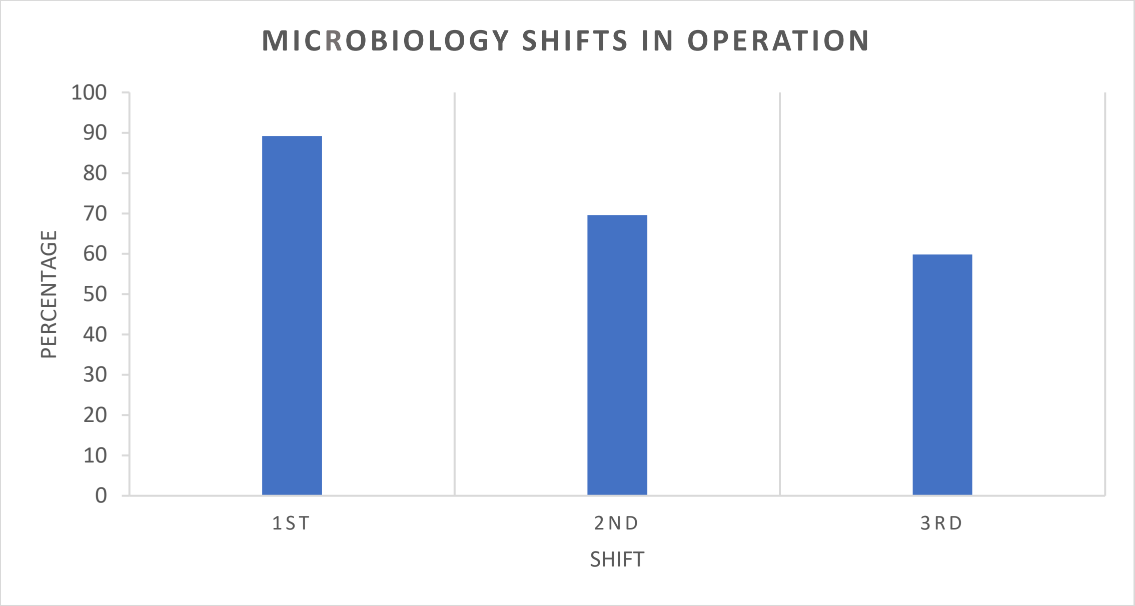

Shift	N	%
1st	173	89.2
2nd	135	69.6
3rd	116	59.8

Q6. Where is your laboratory located?
Reponses were received from 44 states plus the District of Columbia.

	N	%
Arizona	3	1.5
California	8	4.1
Colorado	1	0.5
Connecticut	3	1.5
District of Columbia	1	0.5
Florida	7	3.6
Georgia	3	1.5
Hawaii	1	0.5
Idaho	1	0.5
Illinois	10	5.2
Indiana	6	3.1
Iowa	4	2.1
Kansas	2	1.0
Kentucky	8	4.1
Louisiana	1	0.5
Maine	1	0.5
Maryland	6	3.1
Massachusetts	8	4.1
Michigan	9	4.6
Minnesota	1	0.5
Mississippi	1	0.5
Missouri	3	1.5

	N	%
N. Carolina	7	3.6
N. Dakota	4	2.1
Nebraska	3	1.5
Nevada	1	0.5
New Hampshire	3	1.5
New Jersey	4	2.1
New Mexico	1	0.5
New York	10	5.2
Ohio	9	4.6
Oklahoma	2	1.0
Pennsylvania	11	5.7
Rhode Island	2	1.0
S. Carolina	2	1.0
S. Dakota	1	0.5
Tennessee	4	2.1
Texas	10	5.2
Utah	1	0.5
Vermont	2	1.0
Virginia	7	3.6
Washington	7	3.6
West Virginia	10	5.2
Wisconsin	4	2.1
Wyoming	1	0.5

OVERSITE AND MANAGMENT

Q7. Who has director-level oversight over the microbiology laboratory at your institution?
Of the respondents, 34% indicated an individual with a PhD/DrPH/ScD has director-level oversight over the microbiology laboratory. The remaining 66% indicated that an MD/DO has oversite in some capacity, either full or part time. The wording of the choice MD/PhD Director may have caused confusion; therefore all additional analysis is done looking at MD/DO vs PhD/DrPH/ScD.

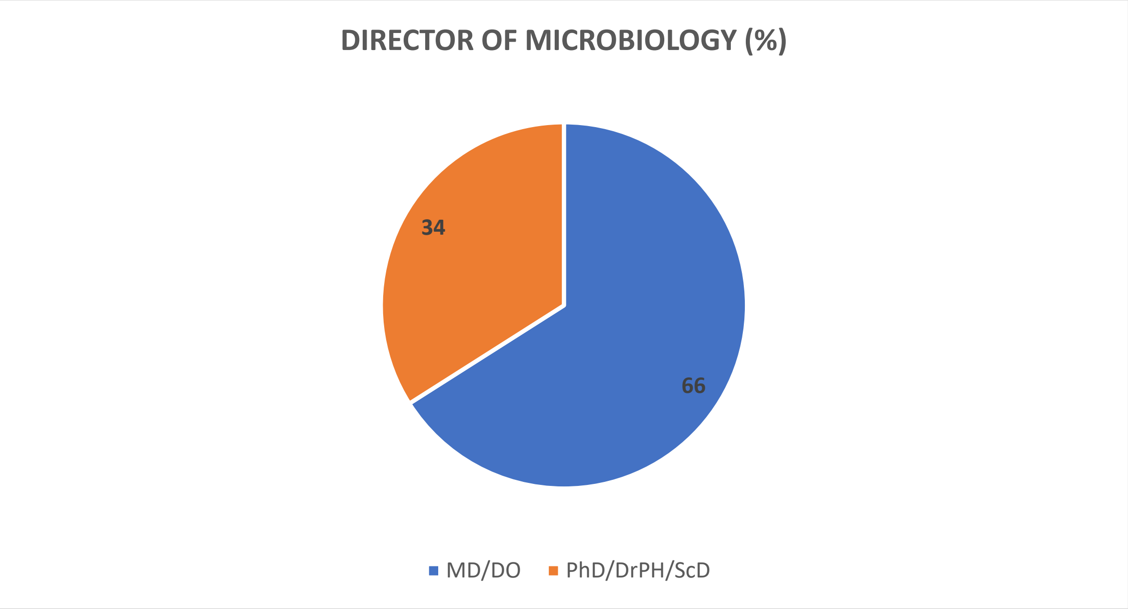

Director of Microbiology	N	%
MD/DO	128	66.0
• MD/DO Director dedicated to Microbiology	32	16.5
• MD/DO Pathologist, oversees multiple lab sections	52	26.8
• MD/PhD Director	44	22.7
PhD/DrPH/ScD Director	66	34.0

Q8. How many supervisors and/or managers are in your microbiology laboratory?
Of the respondents, 48.5% indicated they had one supervisor and/or manager in their respective microbiology laboratory.

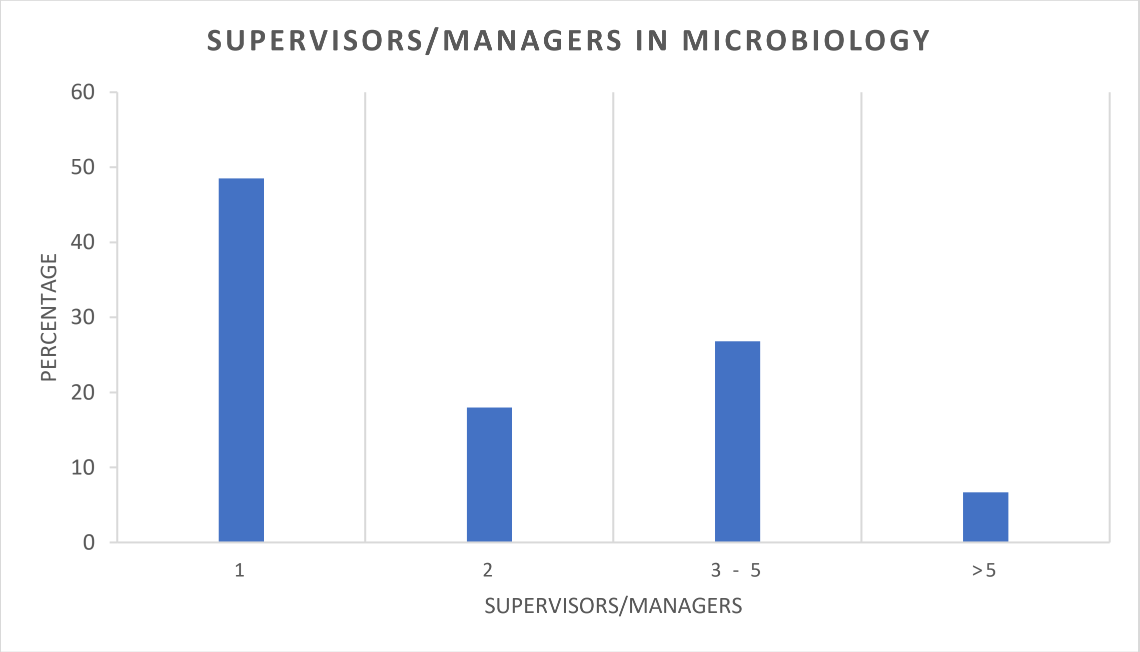

Supervisors/Managers	N	%
1	94	48.5
2	35	18.0
3 - 5	52	26.8
>5	13	6.7

STAFFING AND VACANCIES

Q9. How many Medical Laboratory Scientists (4-year degree or licensed), not including per diem employees, contractors, and supervisory/administrative staff, are employed in your microbiology laboratory?

Of the respondents, 61% indicated there were <25 MLS in the laboratory where they were employed and 39% reported ≥25.

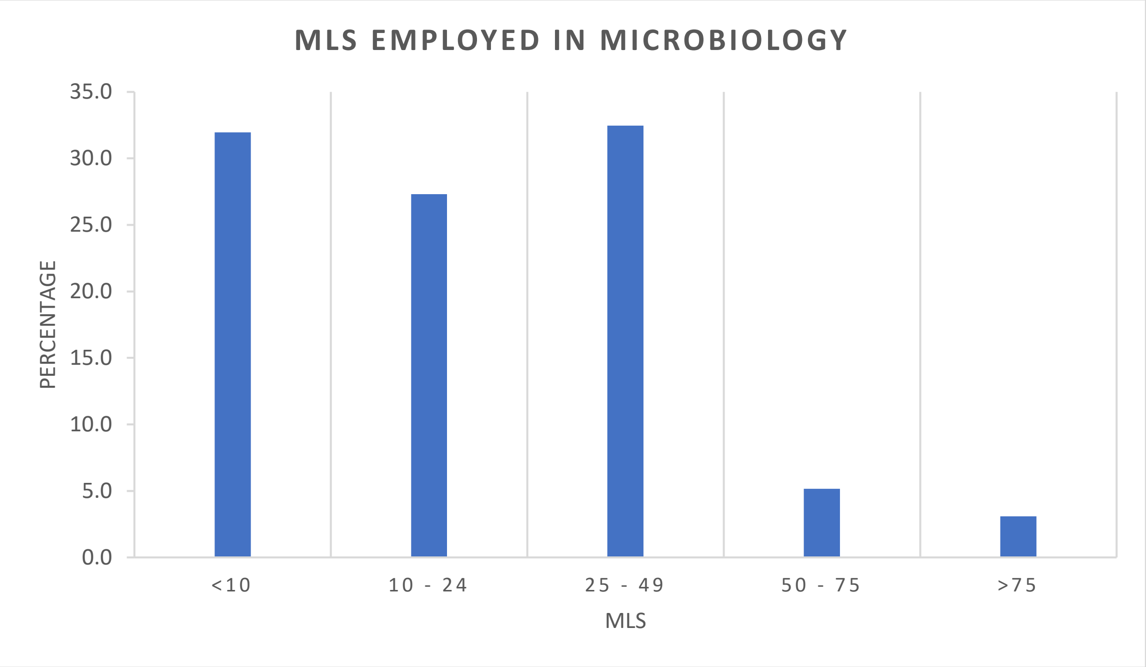

MLS Employed in Microbiology	N	%
<10	62	32.0
10 - 24	53	27.3
25 - 49	63	32.5
50 - 75	10	5.2
>75	6	3.1

Q10. How many Medical Laboratory Technicians (< = 2-year degree), not including per diem employees, contractors, and supervisory/administrative staff, are employed in your microbiology laboratory?

Of the respondents, 78.4% indicated there were less than 10 MLT in the microbiology laboratory.

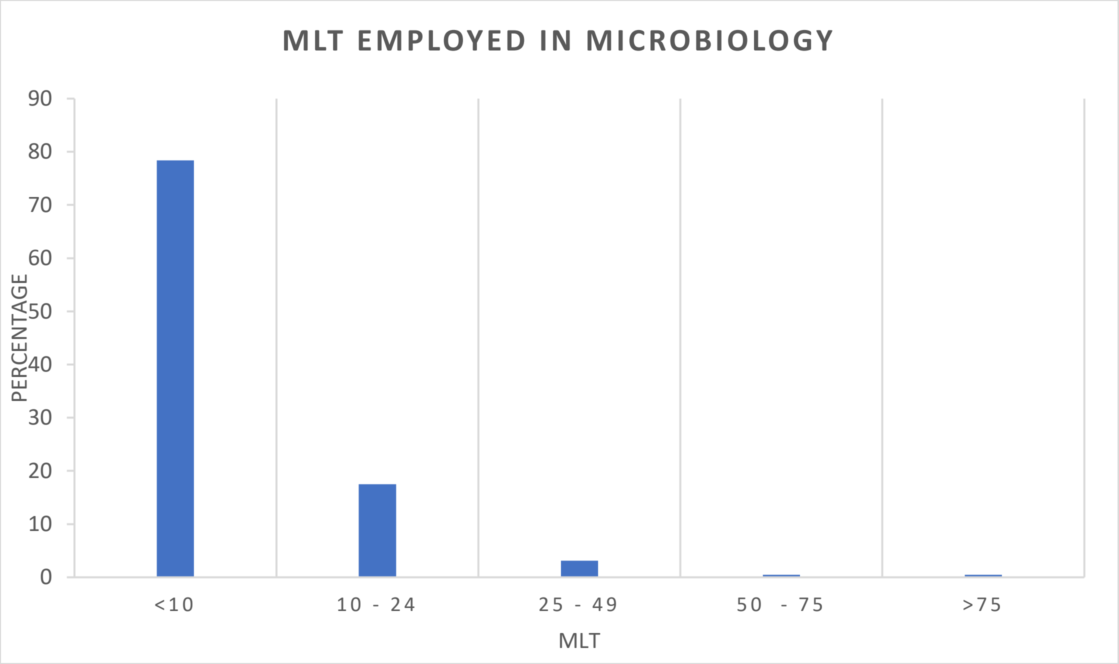

Number of MLT Employed in Microbiology	N	%
<10	152	78.4
10 - 24	34	17.5
25 - 49	6	3.1
50 - 75	1	0.5
>75	1	0.5

Q11. How many open positions are currently available for Medical Laboratory Scientists (4-year degree or licensed) in your microbiology laboratory?
Overall, 80% of respondents reported 1 or more open positions for MLS in microbiology.

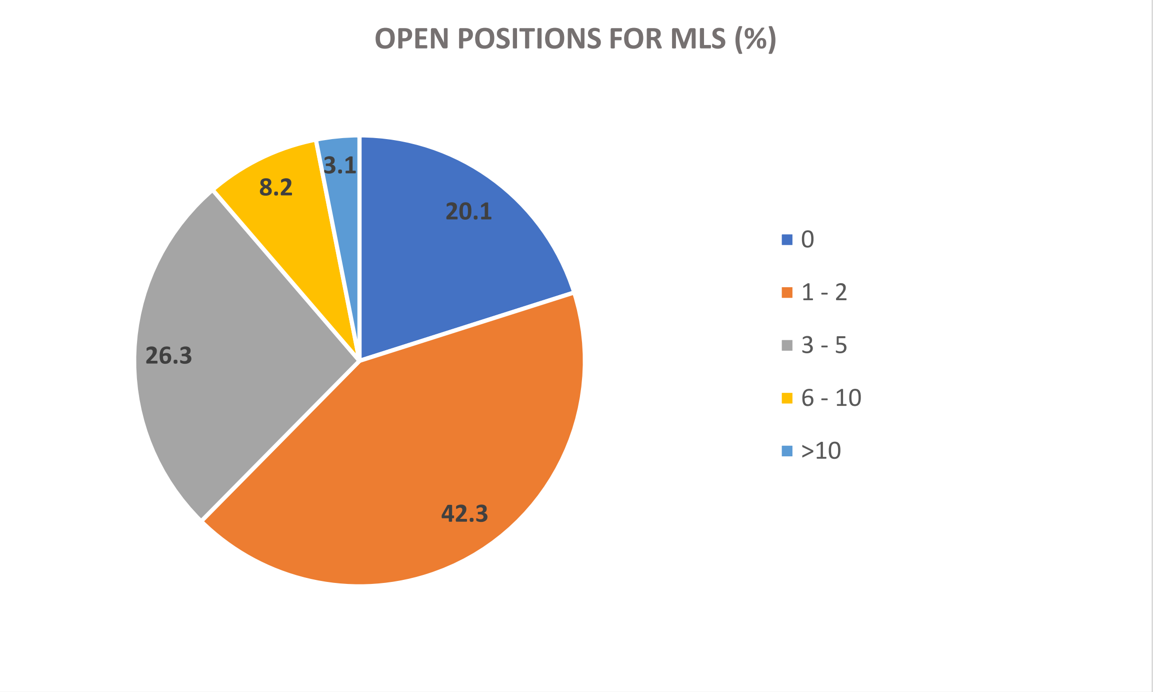

Open Positions for MLS	N	%
0	39	20.1
1 - 2	82	42.3
3 - 5	51	26.3
6 - 10	16	8.2
>10	6	3.1

Q12. How many open positions are currently available for Medical Laboratory Technicians (< = 2-year degree) in your microbiology laboratory?

Of the respondents, 53% indicated there were one or more open positions at the time of the survey.

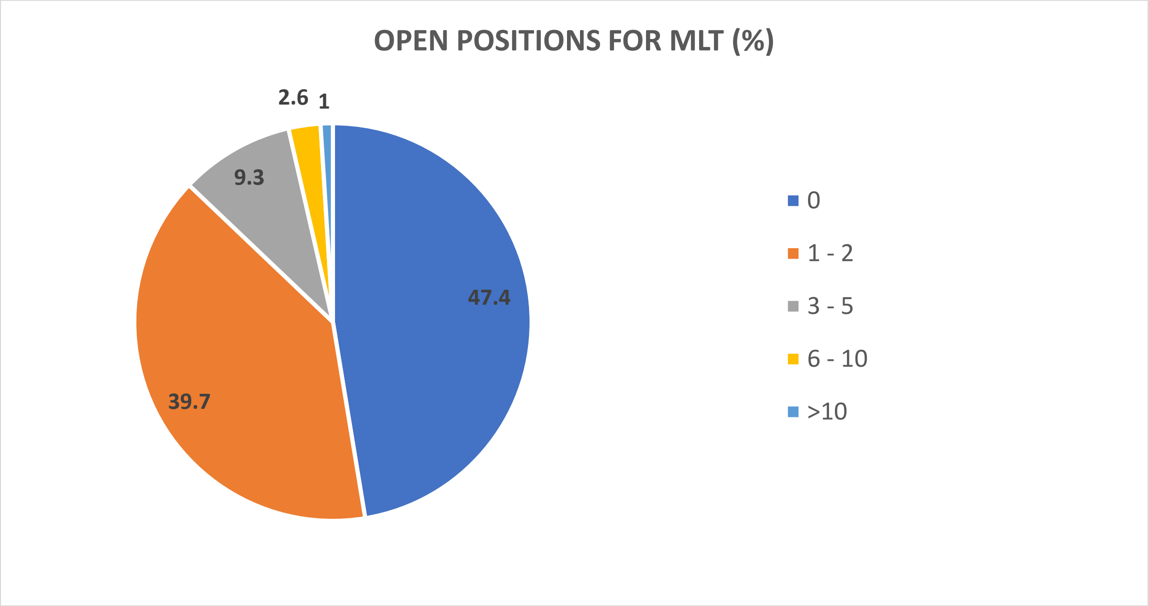

Open positions for MLT	N	%
0	92	47.4
1 - 2	77	39.7
3 - 5	18	9.3
6 - 10	5	2.6
>10	2	1.0

Q13. Once a Medical Laboratory Scientist position is vacant, on average, how long does it take to fill?
Of the respondents, 88 (41.90%) indicated it takes about 4-6 months to fill a vacancy.

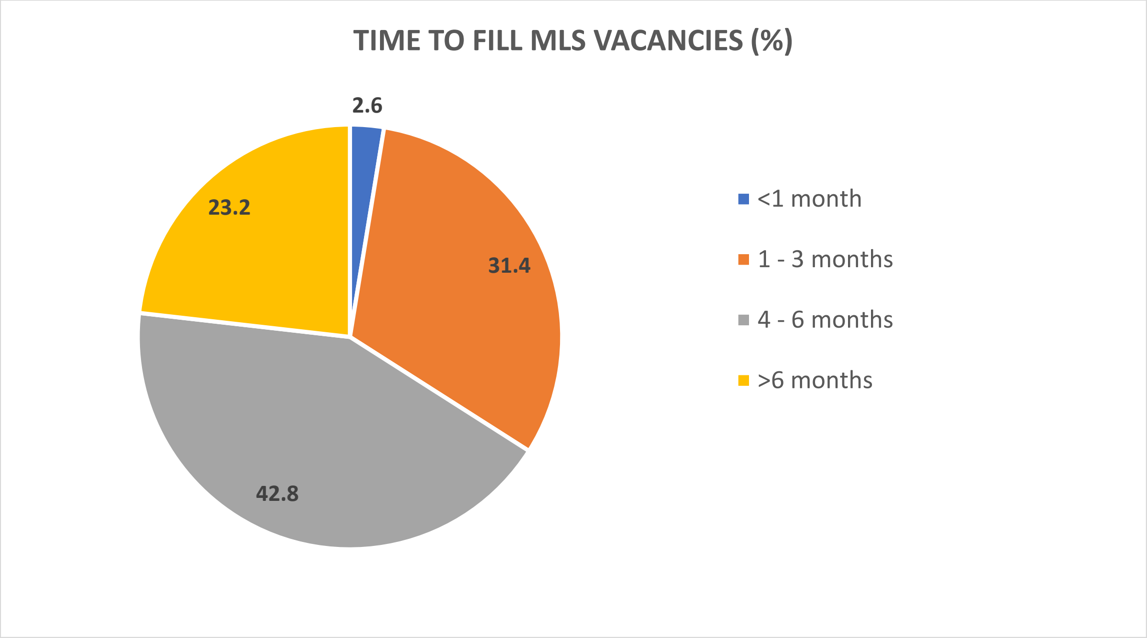

Time to Fill Vacant MLS Position	N	%
<1 month	5	2.6
1 - 3 months	61	31.4
4 - 6 months	83	42.8
>6 months	45	23.2

TRAINING, CERTIFICATION AND CHALLENGES

Q14. At your institution, what is the average amount of time required to train a new Medical Laboratory Scientist in:

Over 65% of respondents indicated it takes at least 3 months to train a MLS to work in Bacteriology. For Molecular Diagnostic, 64% reported training can be accomplished in 1 to 3 months.

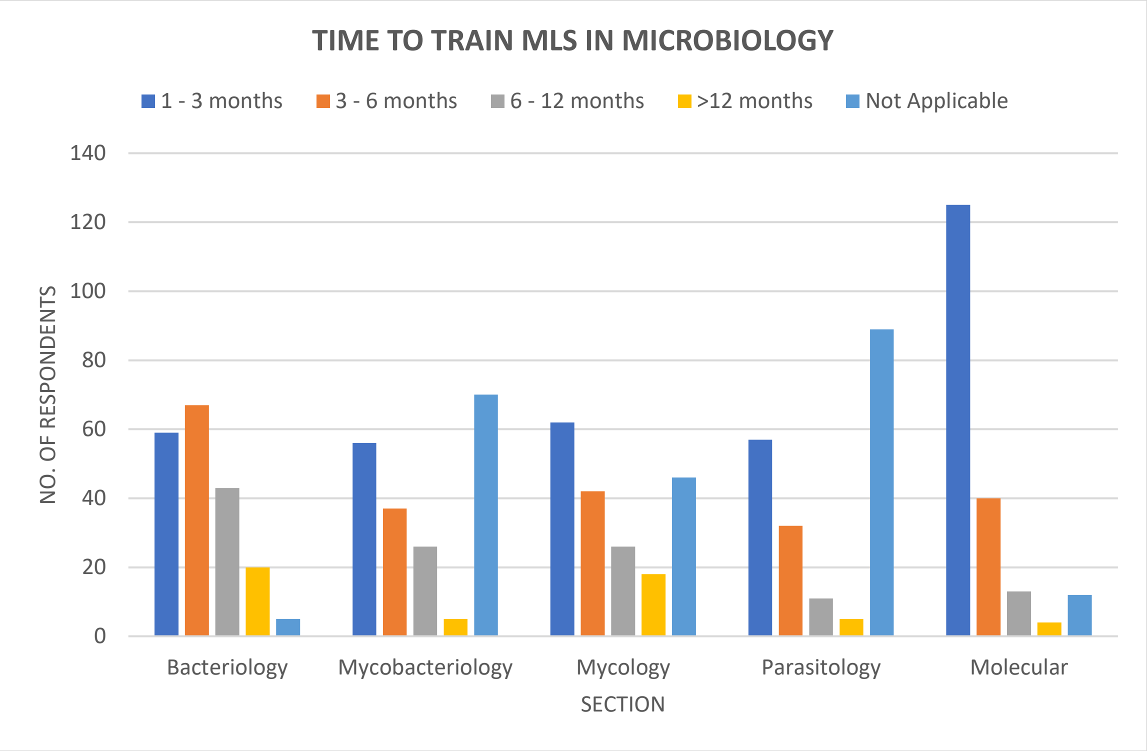

	Section N (%)				
	Bacteriology	Mycobacteriology	Mycology	Parasitology	Molecular
1 - 3 months	59 (30.4)	56 (28.9)	62 (32.0)	57 (29.4)	125 (64.4)
3 - 6 months	67 (34.5)	37 (19.1)	42 (21.6)	32 (16.5)	40 (20.6)
6 - 12 months	43 (22.2)	26 (13.4)	26 (13.4)	11 (5.7)	13 (6.7)
>12 months	20 (10.3)	5 (2.6)	18 (9.3)	5 (2.6)	4 (2.1)
NA	5 (2.6)	70 (36.1)	46 (23.7)	89 (45.9)	12 (6.2)

Abbreviations: NA=Not Applicable

Q15. What are the biggest obstacles to filling the vacant Medical Laboratory Scientists' positions? Rank them from biggest (1) to smallest (5) obstacle.

Of the respondents, 45.4% indicated a lack of qualified applicants was the biggest obstacle to filling a vacancy followed by competition from others (23.7%).

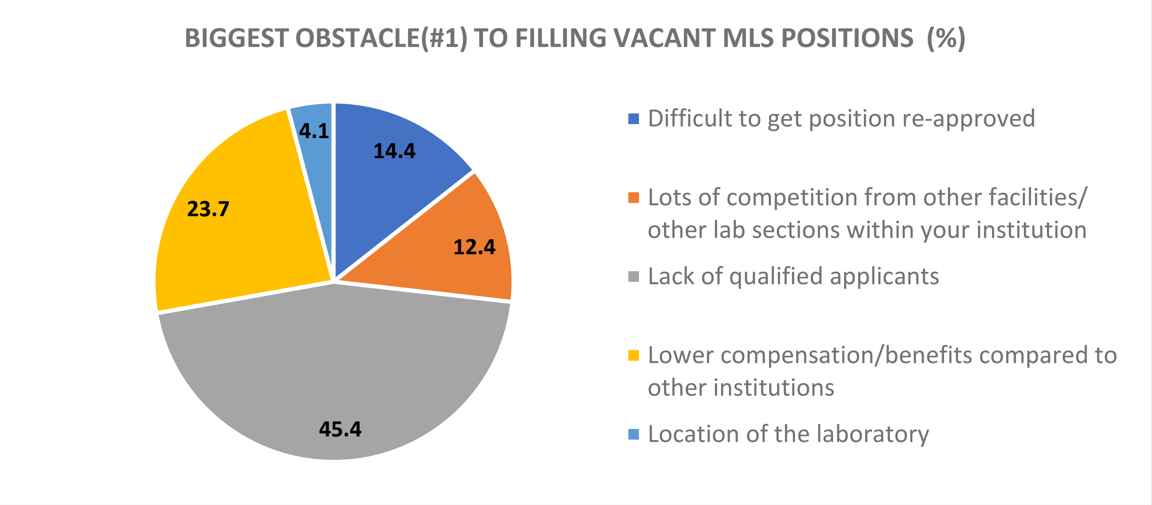

	1	2	3	4	5
Difficult to get position re-approved	28 (14.4)	14 (7.2)	40 (20.6)	51 (26.3)	61 (31.4)
Lots of competition from other facilities/ other lab sections within your institution	24 (12.4)	56 (28.9)	63 (32.5)	33 (17.0)	18 (9.3)
Lack of qualified applicants	88 (45.4)	54 (27.8)	35 (18.0)	13 (6.7)	4 (2.1)
Lower compensation/benefits compared to other institutions	46 (23.7)	51 (26.3)	33 (17.0)	46 (23.7)	18 (9.3)
Location of the laboratory	8 (4.1)	19 (9.8)	23 (11.9)	51 (26.3)	93 (47.9)

Q16. Once a Medical Laboratory Scientist position is vacated, do you have to justify the position before it is posted?

Of the respondents, 72.7% indicated a vacancy does have to be justified prior to advertising the position.

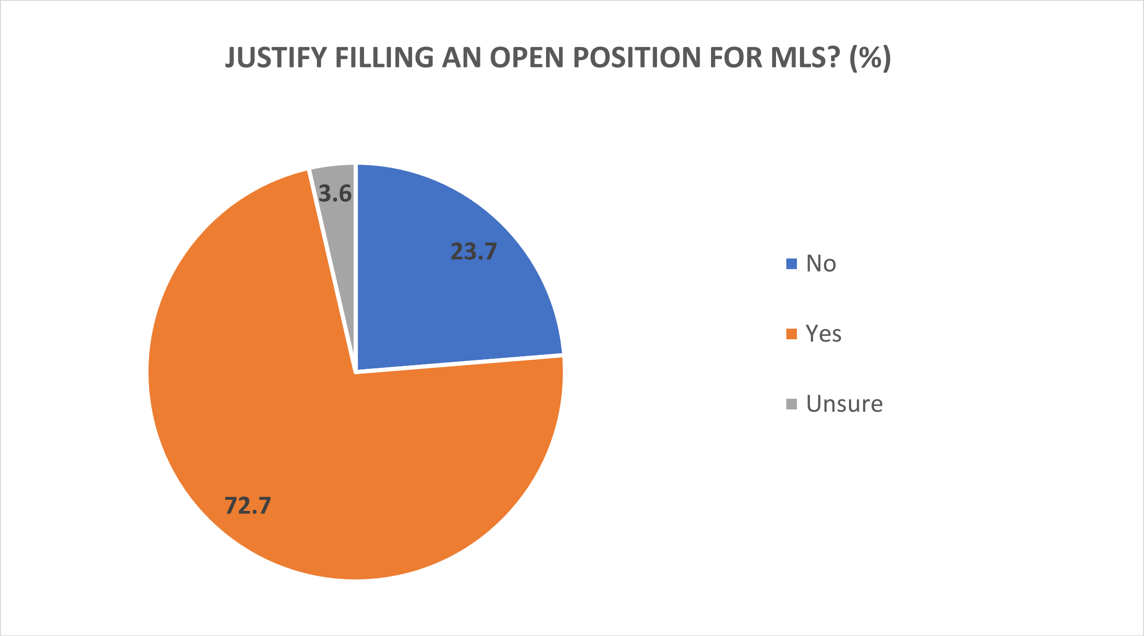

Do you have to justify filling an open position for MLS?	N	%
No	46	23.7
Yes	141	72.7
Unsure	7	3.6

Q17. Does your state or employer require Medical Laboratory Scientists (4-year degree) be certified or certification eligible to perform high complexity testing?

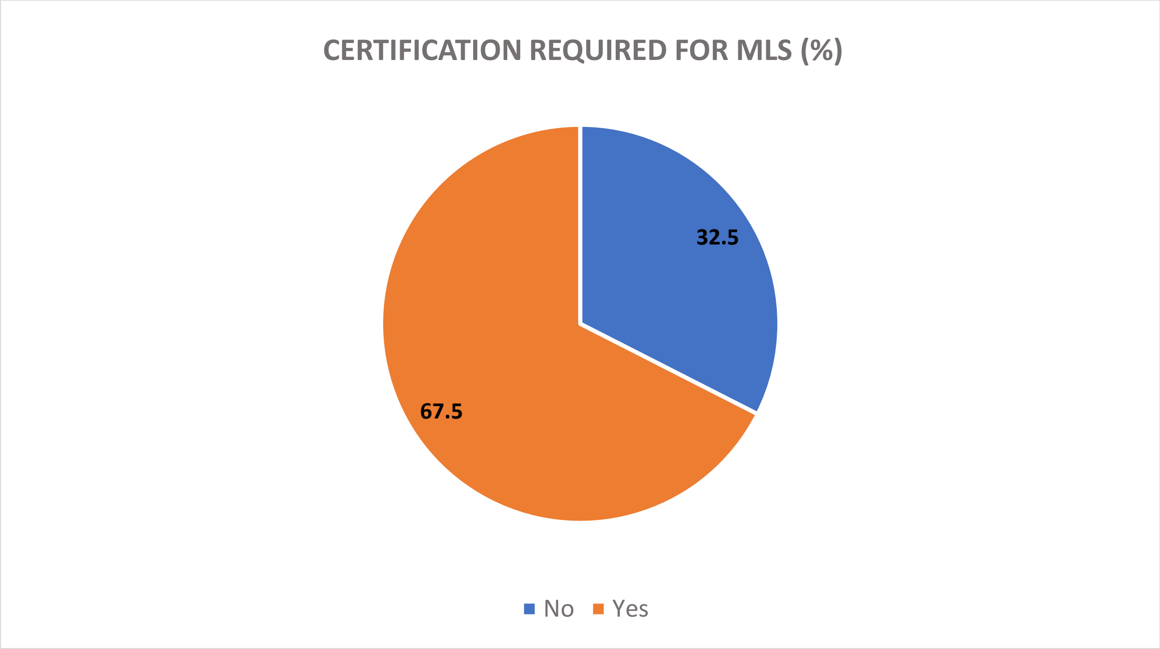

MLS certification required by state or employer?	N	%
No	63	32.5
Yes	131	67.5

Q18. Does your state or employer require Medical Laboratory Technicians (< = 2-year degree) be certified or certification eligible to perform high complexity testing?

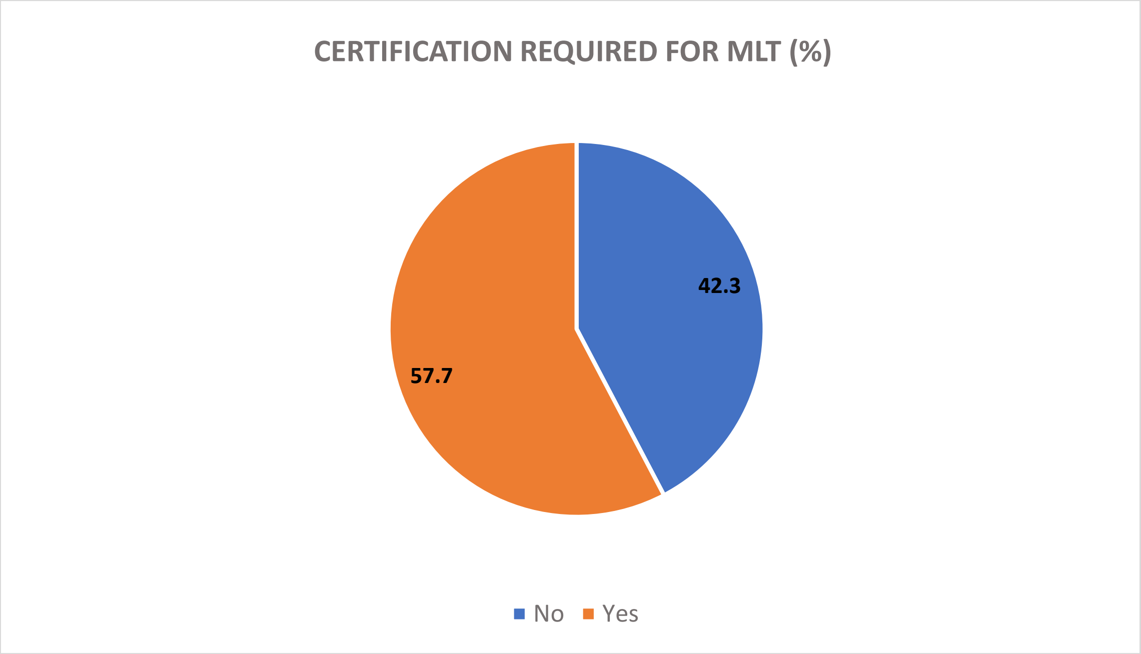

MLT certification required by state or employer?	%	N
No	42.3	82
Yes	57.7	112

Comments

Q19. Please provide any comments or concerns here. (Optional)

The survey allowed an open ended response and select comments are provided below. They have been grouped based on the major theme of the response into four categories: Pay and Compensation, Staffing Challenges, Training Programs and Other Challenges.

**PAY AND COMPENSATION**
Please help us. The salaries are killing this profession.
Retention of the best techs is a problem due to competition to go back to school (PhD, MD, MPH) or to industry/other labs that pay more.
The biggest obstacle is competing with the salary that other institutions pay vs a University setting.
We do have a shortage of qualified applicants but the root of that issue is pay. Lab professionals need to make more money. They need to make the same as nurses with similar education/certification/complexity of work. If pay was better more would want to go in to this field and more training opportunities would follow.
We struggle to retain employees mostly due to lower wages than surrounding areas. The next most common reason for departure is the extremely slow pace of training.
3rd shift techs especially those interested in microbiology can take 6 months or longer to replace, even with $10K sign on bonuses. Local hospital labs are very competitive and offer more incentives to retain any MLS student grads, including tuition forgiveness benefits and starting wages of $27 per hour. We have trained a few qualified college grads from scratch in Microbiology but it can take up to 2 years to learn the basic benches at the same competency level as MLS grads. We encourage them to become ASCP certified in Micro, but there is no way to force them. Senior leadership team including HR does not seem to understand the crisis with future qualified workforce in the lab, yet we continue to get more work from smaller labs who cannot maintain staffing. There is possibly too much faith on automation to buffer the shortage, instead of proactively partnering with MLS programs to recruit and train lab professionals or provide financial support, or reaching out to professional organizations like ASM. Like Cyto and Histo techs, it is time for a Microbiology tech designation with a separate job description and pay scale to better address the shortage of qualified and experienced microbiologists, because even though a tech can be trained on basic bacteriology benches within a year or so, to become competent enough to train others in AFB, Mycology, Parasitology or virology, and to be eligible for microbiology lab leadership positions, much more effort is needed. Those with micro expertise in several sections are extremely valuable, especially for the highly complex hospital microbiology testing, and thus retention must become a priority or those types of tests will eventually need to be sent out. Some lab assistants don't want to work in the microbiology lab because they observe how much more complicated it is than working in other lab sections for the same amount of money, so a more effective career ladder is needed for highly competent microbiology lab assistants as well.
It is very difficult to find MT or MLS. This is a dying field due to the fact pay is so poor compared to nurses. Many forget the hospital doesn't run without the lab.
Training Programs

Our hospital system has an MLS training program, which is really helpful for finding qualified applicants.
School programs for MLS/MLT in the area are closing down, so we often rely on travel agency staffing until we can recruit international candidates.
The Laboratory profession desperately needs more teaching facilities and resources especially in smaller states such as Oklahoma. COVID-19 was somewhat of a help in that Administration was more willing to give "extra" resources and staff positions to the laboratory but as COVID-19 infections decrease I believe this will also decrease.
Need more support from the Federal and State Govt for clinical laboratory training programs. Hiring non-certified personnel for clinical laboratory positions only furthers to degrade and devalue this profession. We need more certified personnel who are trained in QA/QC, PPE use/safety, HIPAA, and diagnostic testing.
Three modes of techs, in order of compensation: 1) Non-registered technicians: We do not have problems finding and attracting qualified BA/BS level scientist come our way. They qualify as non-registered technicians via CAP/CLIA for WA state. We have difficulties retaining them past 2 years because 1) not all want to continue on into lab medicine 2) of those who want to continue, licensure opportunities are challenging. 2) Licensed medical technicians: have historically been successful in attracting MLT (associate's degree + coursework/BA/BS + 1 year MLT degree +licensure). This year is an exception, we've had positions open for months without applicants. These folks have little to no micro background but pick up the basic routine benches quickly, and there is a clear route of progression through the benches. However, the cost is less exogenous clinical microbiology expertise. 3) Licensed MTs: We run into difficulties attracting MTs (BA/BS + MT ASCP or similar) due to dearth of out-of-job candidates and our pay scale. We have/are training internally to challenge M(ASCP), this is enjoyable and good for the culture of the lab but takes years and runs afoul of productivity metrics. Assumptions taken under Question 15: 1-3 months for very basic bacteriology bench. The rest of training will be cumulative. Blood culture bench for example assumes >1 year on other benches. Mycology and mycobacteriology as well.
We are fortunate to be affiliated with several CLS schools for whose students we provide scholarships. In turn these new techs are obligated for work for us for two years. While this is a great avenue for new employees, they come to us with zero (other than their brief clinical rotations) experience which can't be taught rapidly. We've had numerous technologists with 30+ years of experience leave during the past 18 months and that knowledge can't be replaced easily.
We have a CLS program affiliated with VCUHS and draw heavily from that. We also have been very lucky and had good retention over the past couple of years.
We just started MLS program 2 years ago and have hired almost all graduates in our lab, 3 of 16 in Microbiology.
We must get our medical technology programs started again to provide the skilled technologists needed in all areas of the laboratory.
We provide clinical rotations for CLT and MLS students so we are lucky to have that pipeline of new candidates each year.
**STAFFING CHALLENGES**
A couple of thoughts: 1. Temps are thought by administration to be just as good as permanent employees, they do not think about how long it takes to train them. 2. Lack of Administration support in posting and filling positions 3. Lack of vision of using BS degree staff without certification, pay level is at HS degree so most leave quickly 4. Low effort in hospital to retain experienced staff members 5. Lack of understanding amount of non-RVUs work provided by laboratory staff (QC, etc.)

Discussion about shortages for Microbiology has been discussed for at least last 15 years. We were not able to do much but now it is needed more then ever.
I have a lot of older techs retiring, and some younger techs leaving to either go into a different profession all together (i.e.: infection control) or go to Medical School. It seems like almost every other hospital is having a hard time finding qualified candidates. I believe anyone who is looking for a new job right now is not looking at another hospital/ lab job.
Lab staffing levels have not been increased to match the increase in test volume.
NYS requires license - not certification for Clin Lab science= leading to lack of qualified individuals. Salary offered is less compared to similar or less education level requirement. Salary range in comparison deters from students seeking educational opportunities and certification/licensing. Leading to lack if qualified individuals.
One of our biggest hurdles to find techs is to find those interested in working 2nd/3rd shifts. The other issue we find is that there are 4 large hospital systems in our city and we all compete for the available techs.
Unfortunately we are not large enough to rate designated Techs for each dept. We work in all of them but do have 1 Tech that "supervises". I recently stepped down from this position.
We also have high turnover, we can fill our positions begin training them and then they leave. We are understaffed, so we are always training and always filling holes in the schedule which leads to burnout and job dissatisfaction. In my 6.5 years, I've seen 35 techs leave or retire just in microbiology. Our colleagues in Chem and Heme face the same issues. Has ASM reached out to AACC?
We also have several per diem jobs posted for MLA II and Technologists to cover for pto and fmla
We are a small rural health system. We have less than 5 employees trained to work in microbiology. We have been at minimum staffing for a while laboratory wide. It has been difficult to replace staff due to the lack of qualified applicants. We have several biological science degreed people apply, but they do not lack the understanding of clinical laboratory medicine to be competent in the work we perform.
We currently have 8 positions open of 40, 20% vacancy. All positions are approved, and we have no MLS applicants. We're dropping reqs to capture anyone with a bachelor's degree, which is legal but suboptimal and will require extensive training. We've found that such folks either don't like the work and quit, or move on fairly rapidly so intermediate-term retention is quite poor for people who require so long to train. As an academic place, pay is relatively poor vs. any local place that takes MLS applicants, so we also end up providing well-trained people to private hospitals and industry after short retention. We are a union shop, but the union does not advocate for what we learn in exit interviews are the reasons for leaving (pay, hours, and parking in that order). Related, the hospital does not advocate because it's the union's job, and unlike a typical business does not seem to regard cost of training and attrition at all. I wonder if certain kinds of error would draw attention, which I'm hoping we don't encounter with the push toward minimally-qualified new hires. We're seeing a wave of retirements and people transferring for more pay post-COVID as people feel less risk averse and/or sick of overwork. And, like other places, we had an MLS training program until a few years ago, but that was stopped because "too expensive". That was a nice pipeline, and we didn't have critical staffing shortages when it was active (though turnover was still high, having a new crop every year mitigated).
We have had at least one microbiology position open almost continually for 12 years. One person will retire or move and we start over. In 12 years I have been fully staffed a total of 23 months and not more than 1 year continuously.

We have started to hire techs with BS degree microbiology/immunology as CLA, train them and they need to sit for a certification to go any further
While we do not have open positions in Microbiology, we do in the main lab which provides off-shift coverage.
Department currently without micro technical coordinator. Position posted for >18 months. No qualified candidates with experience
Finding those that have the passion for Microbiology and want to be involved in antibiotic stewardship is a challenge.
Length of training time required to become proficient in the various disciplines exacerbates the challenges around turnover and retention. A lot of investment (time, knowledge, etc.) walks out the door when someone resigns. This revolving door is even more impactful when it is someone who is finally settling in with 2-3 years of experience.
**TRAINING PROGRAMS**
Our hospital system has an MLS training program, which is really helpful for finding qualified applicants.
School programs for MLS/MLT in the area are closing down, so we often rely on travel agency staffing until we can recruit international candidates.
The Laboratory profession desperately needs more teaching facilities and resources especially in smaller states such as Oklahoma. COVID-19 was somewhat of a help in that Administration was more willing to give "extra" resources and staff positions to the laboratory but as COVID-19 infections decrease I believe this will also decrease.
Need more support from the Federal and State Govt for clinical laboratory training programs. Hiring non-certified personnel for clinical laboratory positions only furthers to degrade and devalue this profession. We need more certified personnel who are trained in QA/QC, PPE use/safety, HIPAA, and diagnostic testing.
Three modes of techs, in order of compensation: 1) Non-registered technicians: We do not have problems finding and attracting qualified BA/BS level scientist come our way. They qualify as non-registered technicians via CAP/CLIA for WA state. We have difficulties retaining them past 2 years because 1) not all want to continue on into lab medicine 2) of those who want to continue, licensure opportunities are challenging. 2) Licensed medical technicians: have historically been successful in attracting MLT (associate's degree+coursework/BA/BS + 1 year MLT degree +licensure). This year is an exception, we've had positions open for months without applicants. These folks have little to no micro background but pick up the basic routine benches quickly, and there is a clear route of progression through the benches. However, the cost is less exogenous clinical microbiology expertise. 3) Licensed MTs: We run into difficulties attracting MTs (BA/BS + MT ASCP or similar) due to dearth of out-of-job candidates and our payscale. We have/are training internally to challenge M(ASCP), this is enjoyable and good for the culture of the lab but takes years and runs afoul of productivity metrics. Assumptions taken under Question 15: 1-3 months for very basic bacteriology bench. The rest of training will be cumulative. Blood culture bench for example assumes >1 year on other benches. Mycology and mycobacteriology as well.
We are fortunate to be affiliated with several CLS schools for whose students we provide scholarships. In turn these new techs are obligated for work for us for two years. While this is a great avenue for new employees, they come to us with zero (other than their brief clinical rotations) experience which can't be taught rapidly. We've had numerous technologists with 30+ years of experience leave during the past 18 months and that knowledge can't be replaced easily.
We have a CLS program affiliated with VCUHS and draw heavily from that. We also have been very lucky and had good retention over the past couple of years.

We just started MLS program 2 years ago and have hired almost all graduates in our lab, 3 of 16 in Microbiology.
We must get our medical technology programs started again to provide the skilled technologists needed in all areas of the laboratory.
We provide clinical rotations for CLT and MLS students so we are lucky to have that pipeline of new candidates each year.
**OTHER/MULTIPLE CHALLENGES**
Currently our lab has no laboratory director as it is difficult to fill that position. we rely on a "borrowed" pathologist to fulfill our CLIA director obligations. Our laboratory does not require any certifications for microbiologists and we usually have quite a few very qualified candidates who have majored in public health or biochemistry who have trained and have become excellent molecular or general microbiologists. I think if we required certification it would be very hard to be competitive with hospital labs
Lack of affordable housing in our rural area is a big issue. We chronically lose young technologists to big cities that have more opportunities and more cultural activities. Our younger staff often see the profession of medical laboratory science as a stepping stone to something else, and not as a life-long career path with room for growth.
Shortage of Mts is a huge problem and will not get better until Mts take control of their issues. The ASCP is of little use to them.
The hardest part is getting positions reapproved and justifying the need, increasing work load and regulatory requirements/paperwork do not make a difference. We have excellent technologists apply but no ability to hire.
The quality of applicants is also declining. It appears we spend more time trying to meet regulatory requirements than focusing on training and competency due to the potential repercussions and sanitations. Spending so much administrative time in daily tasks has not allowed for adequate training and staffing is already short and it is difficult to explain to administration why the productivity metrics (billable tests per month) look lower, yet we still feel the strain of short staffing. We need good, quality metrics and an education of administrators on how Lab is different than may other hospital services. I wish I could have a person dedicated to quality assurance but the hospital I am at does not see the values and lumps in these functions with the hospital QA manager. This QA manager is an RN and never trained in lab.
There has been a substantial number of resignations within the last 2 months, and the salary rate here for a technologist or lab assistant is not nearly as competitive as the hospital next door. Therefore, it has been a challenge to fill the roles, although our hospital is reputable. The current morale in the Micro Lab is low. There is too much pressure from providers and the hospital to (1) continue with routine Micro testing, (2) perform SARS-CoV-2 testing and keep up with complicated workflows (plus incessant calls), (3) stay on top of intermittent supply shortage issues, (4) support research projects as they ramp up again, and (5) bring more tests online as part of a hospital recovery plan.

ADDITIONAL ANALYSIS:

Based on answers to the core questions, additional evaluation was done for comparative analysis of staffing, supervision, volumes, and vacancies.

A1. Annual Test Volume versus Laboratory Type

The most common response to this question across all lab types was 200,000- 499,999 test/year from 51(26.3%) labs followed by 500,000-1,000,000 from 34 (17.5%) labs. The least common response was <5,000 from 5 (2.5%) labs.

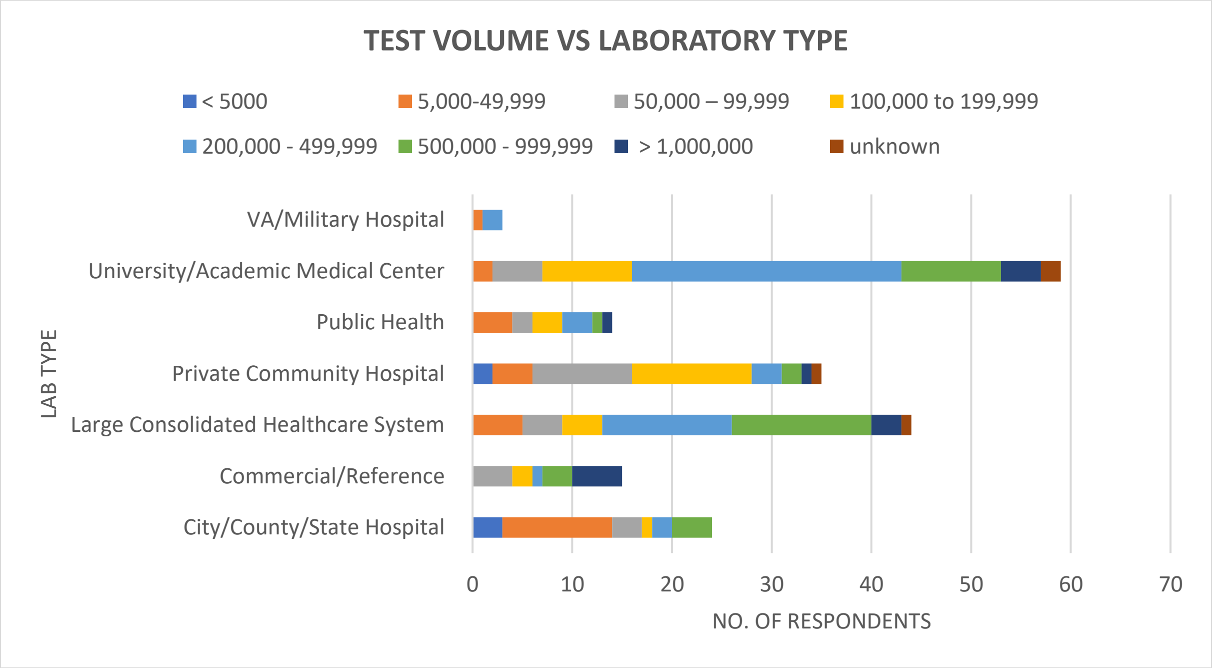

	Test Volume N (% by Lab Type)							
Type of Laboratory	< 5000	5,000 - 49,~~000~~999	50,000 – 99,999	100,000 - 199,999	200,000 - 499,999	500,000 - 999,999	> 1,000,000	unknown
City/County/State Hospital n=24	3 (12.5)	11 (45.8)	3 (12.5)	1 (4.2)	2 (8.3)	4 (16.7)	0 (0)	0 (0)
Commercial/Reference n=15	0 (0)	0 (0)	4 (26.7)	2 (13.3)	1 (6.7)	3 (20.0)	5 (33.3)	0 (0)
Large Consolidated Healthcare System n=44	0 (0)	5 (11.4)	4 (9.1)	4 (9.1)	13 (29.5)	14 (31.8)	3 (6.8)	1 (2.3)
Private Community Hospital n=35	2 (5.7)	4 (11.4)	10 (28.6)	12 (34.3)	3 (8.6)	2 (5.7)	1 (2.9)	1 (2.9)
Public Health n=14	0 (0)	4 (28.6)	2 (14.3)	3 (21.4)	3 (21.4)	1 (7.1)	1 (7.1)	0 (0)
University/Academic Medical Center n=59	0 (0)	2 (3.4)	5 (8.5)	9 (45.8)	27 (45.8)	10 (16.9)	4 (6.8)	2 (3.4)
VA/Military Hospital n=3	0 (0)	1 (33.3)	0 (0)	0 (0)	2 (66.7)	0 (0)	0 (0)	0 (0)

A2. Number of Supervisors/Managers versus Laboratory Type
The most common response to this question across all lab types was 1 supervisor/manager from 94 (48.5%) labs followed by 3-5 supervisors/managers from 52 (26.8%) labs. The least common response was >5 from 13 (6.7%)

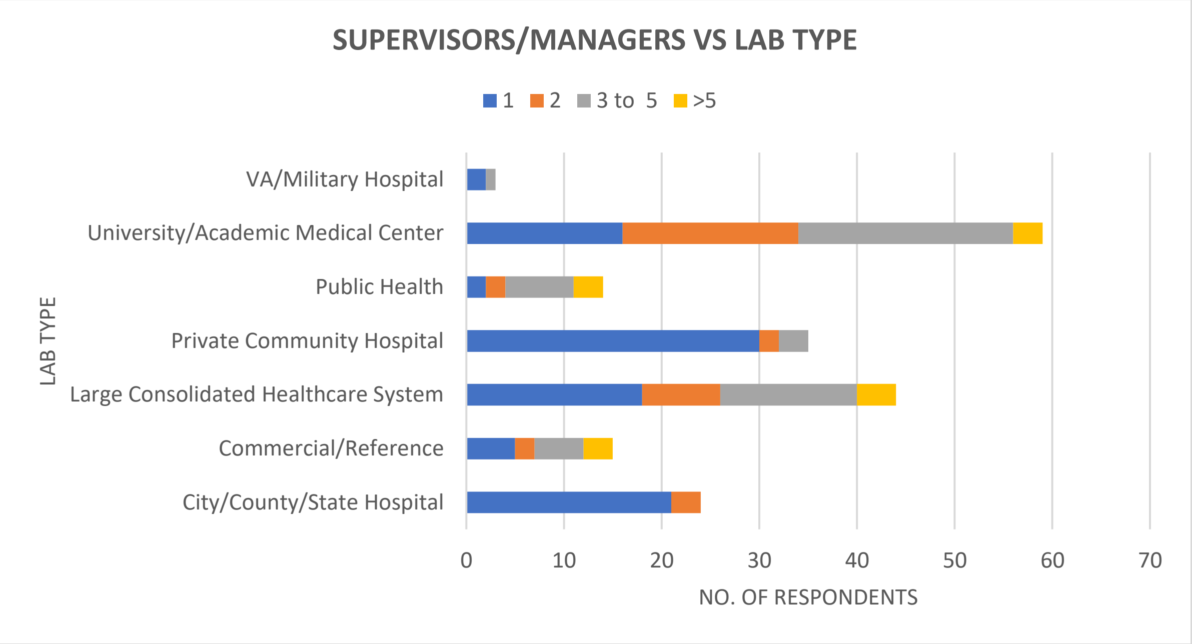

	Supervisors/Managers N(% by Lab Group)			
Type of Laboratory	1	2	3 - 5	>5
City/County/State Hospital (n=24)	21 (87.5)	3 (21.5)	0 (0)	0 (0)
Commercial/Reference (n=15)	5 (33.3)	2 (13.3)	5 (33.3)	3 (20.0)
Large Consolidated Healthcare System (n=44)	18 (40.9)	8 (18.2)	14 (31.8)	4 (9.1)
Private Community Hospital (n=35)	30 (85.7)	2 (5.7)	3 (8.6)	0 (0)
Public Health (n=14)	2 (14.3)	2 (14.3)	7 (50.0)	3 (21.4)
University/Academic Medical Center (n=59)	16 (27.1)	18 (30.5)	22 (37.3)	3 (5.1)
VA/Military Hospital (n=3)	2 (66.7)	0 (0)	1 (33.3)	0 (0)

A3. Number of Supervisors/Managers versus Annual Test Volume
There is a general trend with higher test volumes associated with more supervisors/managers. The most common response was 1 supervisor/manager from 94 (48.5%) of labs.

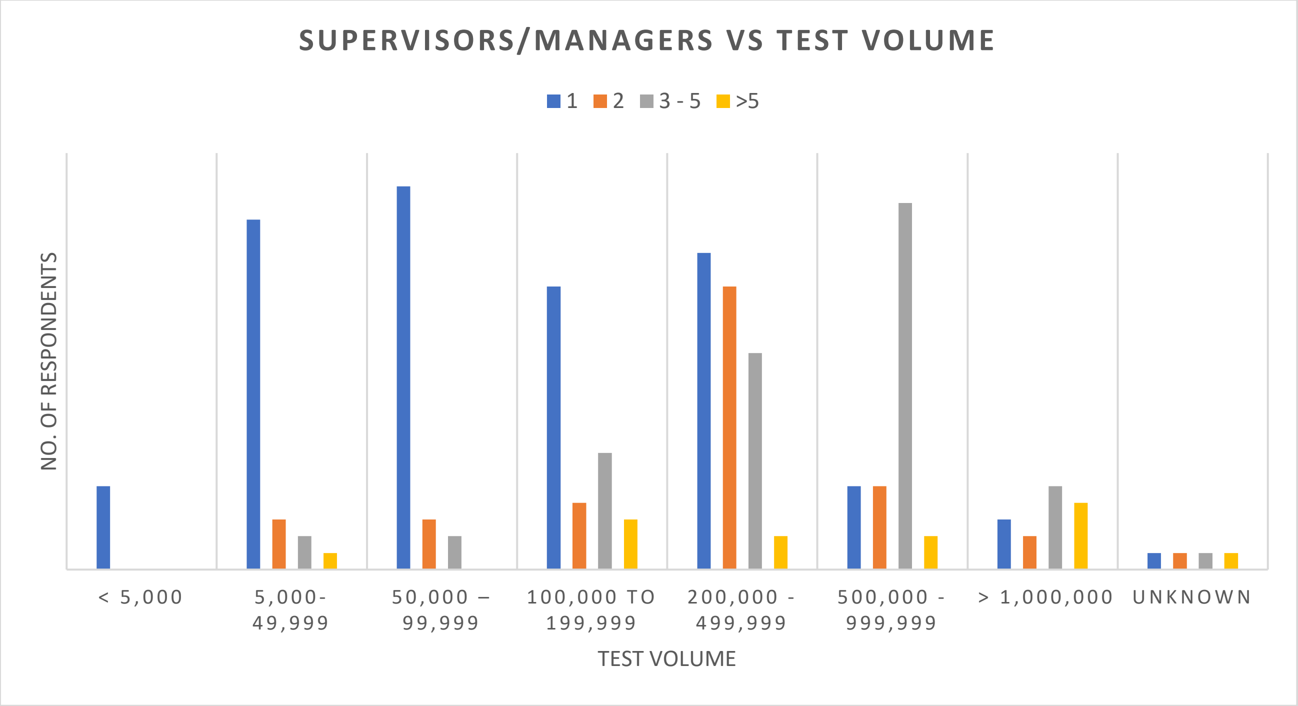

	Supervisors/Managers N(% of volume group)			
Annual Test Volume	1	2	3 - 5	>5
< 5,000 (n=5)	5 (100)	0 (0)	0 (0)	0 (0)
5,000-49,999 (n=27)	21 (77.8)	3 (11.1)	2 (7.4)	1 (3.7)
50,000 – 99,999 (n=28)	23 (82.1)	3 (10.7)	2 (7.1)	0 (0)
100,000 to 199,999 (n=31)	17 (54.8)	4 (12.9)	7 (22.6)	3 (9.7)
200,000 - 499,999 (n=51)	19 (37.3)	17 (33.3)	13 (25.5)	2 (3.9)
500,000 - 999,999 (n=34)	5 (14.7)	5 (14.7)	22 (64.7)	2 (5.9)
>1,000,000 (n=14)	3 (21.4)	2 (14.3)	5 (35.7)	4 (28.6)
Unknown (n=4)	1 (25.0)	1 (25.0)	1 (25.0)	1 (25.0)

A4. Number of Supervisors/Managers versus Medical Laboratory Scientists

There is a general trend with labs with higher test volumes having more supervisors/managers. The most common response was 1 supervisor/manager from 94 (48.5%) of labs. Starting at >24 employees, the most common response was 3 -5 supervisors/managers.

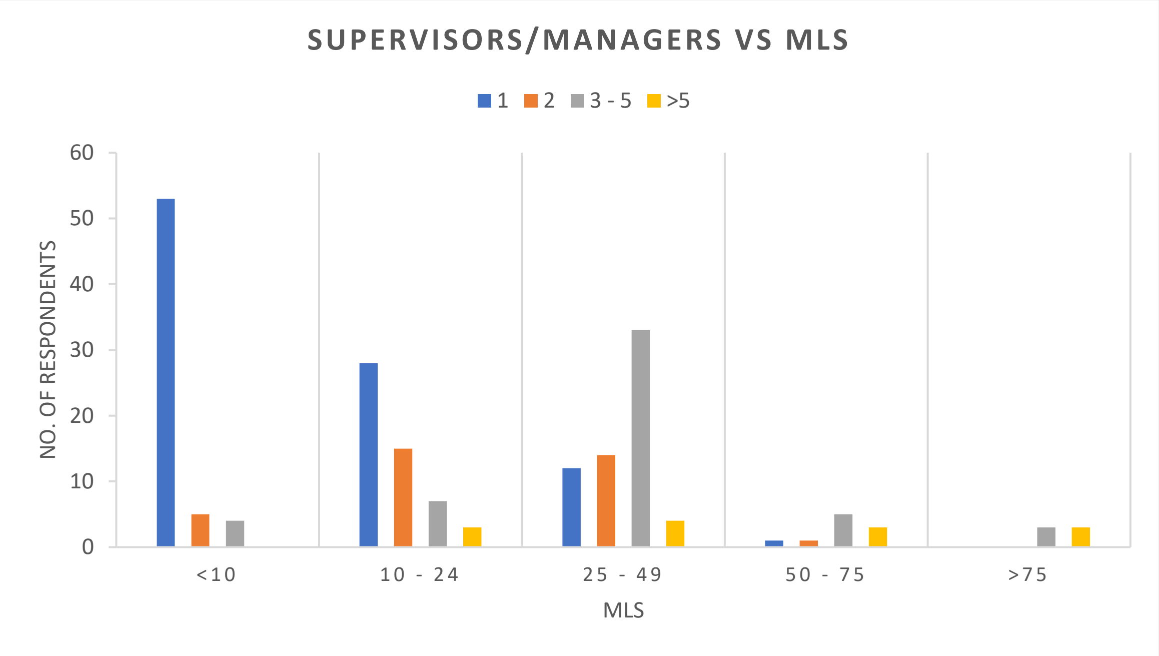

	Supervisors/Managers N(% of MLS group)			
No. of MLS	1	2	3 - 5	>5
<10 (n=62)	53 (85.5)	5 (8.1)	4 (6.5)	0 (0)
10 – 24 (n=53)	28 (52.8)	15 (28.3)	7 (13.2)	4 (5.7)
25 – 49 (n=63)	12 (19.0)	14 (22.2)	33 (52.4)	3 (6.3)
50 – 75 (n=10)	1 (10.0)	1 (10.0)	5 (50.0)	3 (30.0)
>75 (n=6)	0 (0)	0 (0)	3 (50.0)	3 (50.0)

A5. -Number of Medical Laboratory Scientists versus Laboratory Type

There were a wide range of answers to the number of MLS employed for each lab type. The majority of City/County/State Hospitals and Private Community Hospitals reported <25 employees while Academic Medical Centers and Large Health Care Systems reported >25 employees most often.

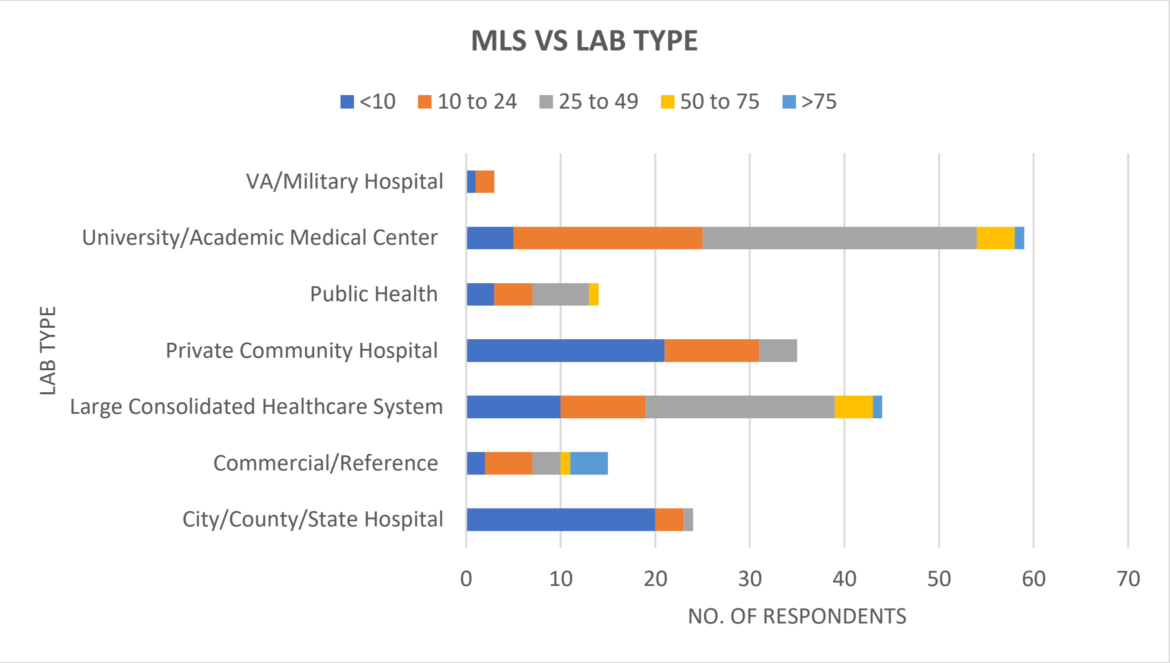

	Medical Laboratory Scientists N(% of lab group)				
Type of Laboratory	<10	10 - 24	25 - 49	50 - 75	>75
City/County/State Hospital (n=24)	20 (83.3)	3 (12.5)	1 (4.2)	0 (0)	0 (0)
Commercial/Reference (n=15)	2 (13.3)	5 (33.3)	3 (20.0)	1 (6.7)	4 (26.7)
Large Consolidated Healthcare System (n=44)	10 (22.7)	9 (20.5)	20 (45.5)	4 (9.1)	1 (2.3)
Private Community Hospital (n=35)	21 (60.0)	10 (28.6)	4 (11.4)	0 (0)	0 (0)
Public Health (n=14)	3 (21.4)	4 (28.6)	6 (42.9)	1 (7.1)	0 (0)
University/Academic Medical Center (n=59)	5 (8.5)	20 (33.9)	29 (49.2)	4 (6.8)	1 (1.7)
VA/Military Hospital (n=3)	1 (33.3)	2 (67.7)	0 (0)	0 (0)	0 (0)

A6. Number of Medical Laboratory Scientists versus Annual Test Volume

For labs with volumes <200,000, the most common response was <10 MLS while for those with volumes >200,000 reported >10.

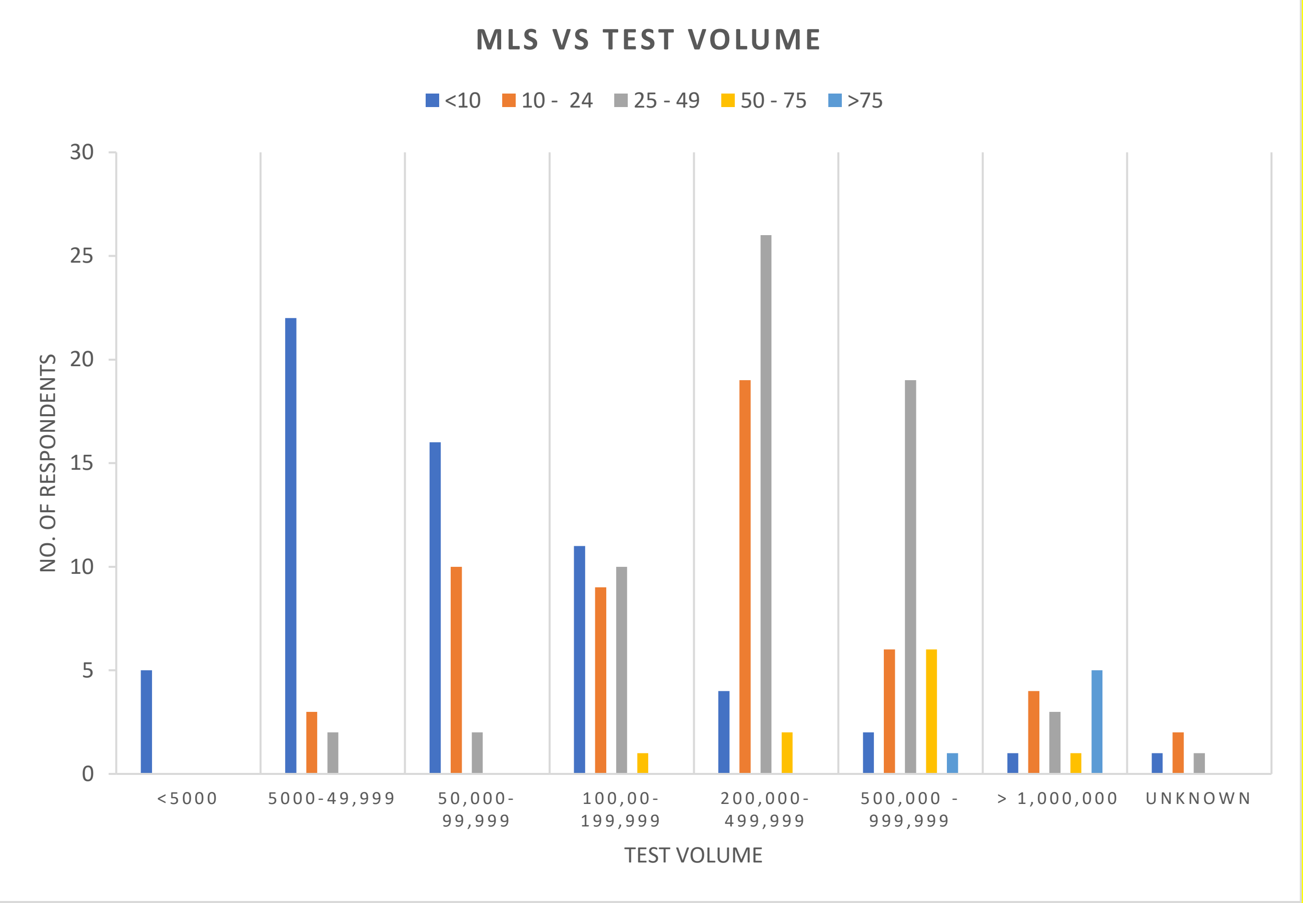

	Annual Test Volume N(% of MLS group)							
No. of MLS	<5000	5000-49,999	50,000-99,999	100,000-199,999	200,000-499,999	500,000-999,999	>1,000,000	unknown
<10 (n=65)	5 (8.1)	22 (35.5)	16 (25.8)	11 (17.7)	4 (6.5)	2 (3.2)	1 (1.6)	1 (1.6)
10 - 24 (n=54)	0 (0)	3 (5.7)	10 (18.9)	9 (17.0)	19 (35.8)	6 (11.3)	4 (7.5)	2 (3.8)
25 - 49 (n=59)	0 (0)	2 (3.2)	2 (3.2)	10 (15.9)	26 (41.3)	19 (30.2)	3 (4.8)	1 (1.6)
50 - 75 (n=10)	0 (0)	0 (0)	0 (0)	1 (10.0)	2 (20.0)	6 (60.0)	1 (10.0)	0 (0)
>75 (n=6)	0 (0)	0 (0)	0 (0)	0 (0)	0 (0)	1 (16.7)	5 (83.3)	0 (0)

A7. Number of Medical Laboratory Technicians versus Laboratory Type

In this survey, the number of MLT employed in Microbiology is lower than the number of MLS. The most common response regardless of lab type was <10. The survey did not allow a response of none so we cannot determine the number of labs that do not employee any MLTs.

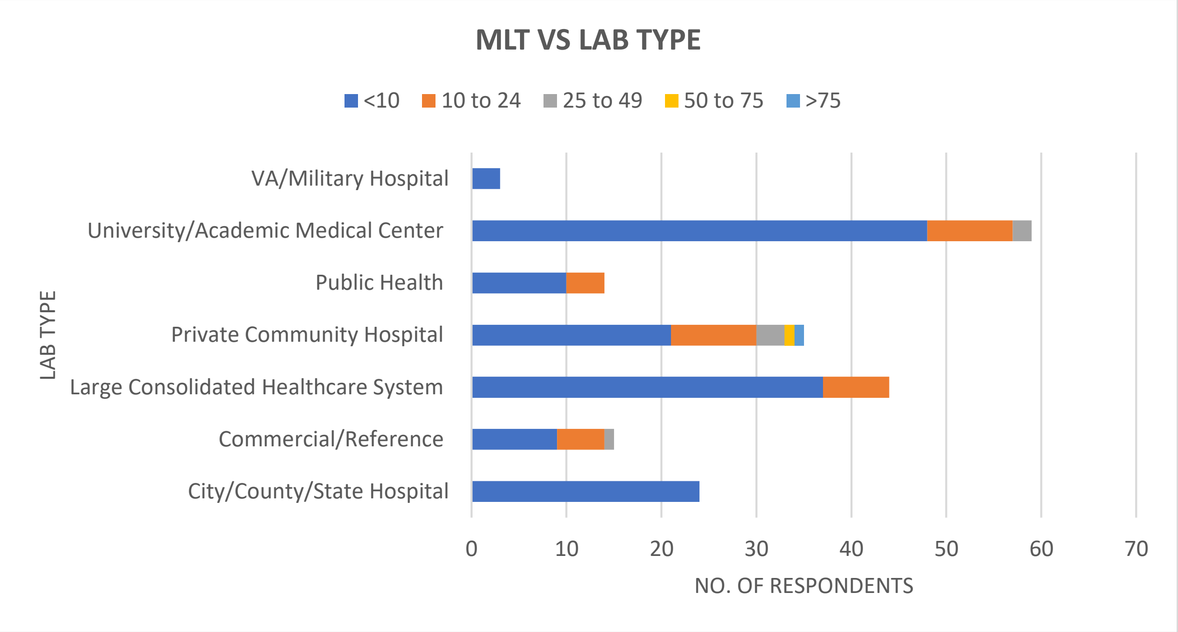

	Number of MLT N(% of lab group)				
Type of Laboratory	<10	10 - 24	25 - 49	50 - 75	>75
City/County/State Hospital (n=24)	24 (100)	0 (0)	0 (0)	0 (0)	0 (0)
Commercial/Reference (n=15)	9 (60.0)	5 (33.3)	1 (6.7)	0 (0)	0 (0)
Large Consolidated Healthcare System (n=44)	37 (84.1)	7 (15.9)	0 (0)	0 (0)	0 (0)
Private Community Hospital (n=35)	21 (60.0)	9 (25.7)	3 (8.6)	1 (2.9)	1 (2.9)
Public Health (n=14)	10 (71.4)	4 (28.6)	0 (0)	0 (0)	0 (0)
University/Academic Medical Center (n=59)	48 (81.4)	9 (15.3)	2 (3.4)	0 (0)	0 (0)
VA/Military Hospital (n=3)	3 (100)	0 (0)	0 (0)	0 (0)	0 (0)

A8. Number of Medical Laboratory Technicians versus Annual Test Volume
The number of MLT employed showed a trend with labs with higher volumes (>200,000) employing more than those with lower volume (<200,000).

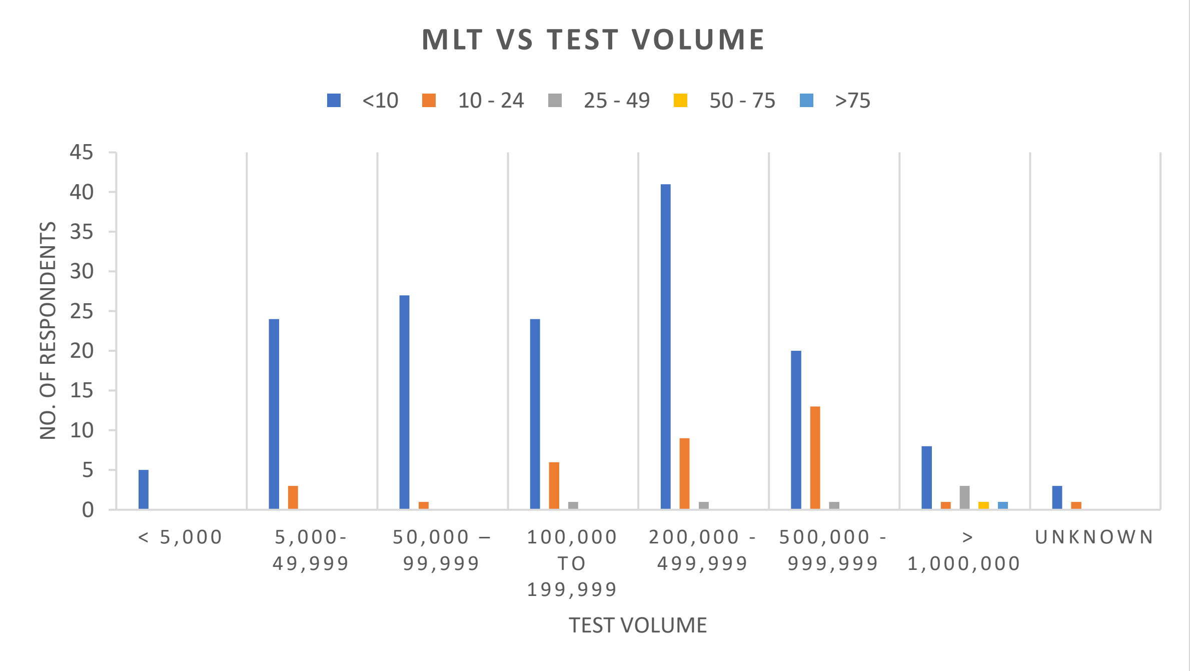

	Annual Test Volume N (% of MLT group)							
# of MLT	<5000	5000-49,999	50,000-99,999	100,000-199,999	200,000-499,999	500,000 - 999,999	>1,000,000	unknown
<10 (n=152)	5 (3.3)	24 (15.8)	27 (17.8)	24(15.8)	41 (27.0)	20 (13.2)	8 (5.3)	3 (2.0)
10 - 24 (n=34)	0 (0)	3 (8.8)	1 (2.9)	6 (17.6)	9 (26.5)	13(38.2)	1 (2.9)	1 (2.9)
25 - 49 (n=6)	0 (0)	0 (0)	0 (0)	1 (16.7)	1 (16.7)	1 (16.7)	3 (50.0)	0 (0)
50 -7 5 (n=1)	0 (0)	0 (0)	0 (0)	0 (0)	0 (0)	0 (0)	1 (100)	0 (0)
>75 (n=1)	0 (0)	0 (0)	0 (0)	0 (0)	0 (0)	0 (0)	1 (100)	0 (0)

A9. Number of Vacancies for Medical Laboratory Scientists versus Laboratory Type

Vacancies are seen across all laboratory types.

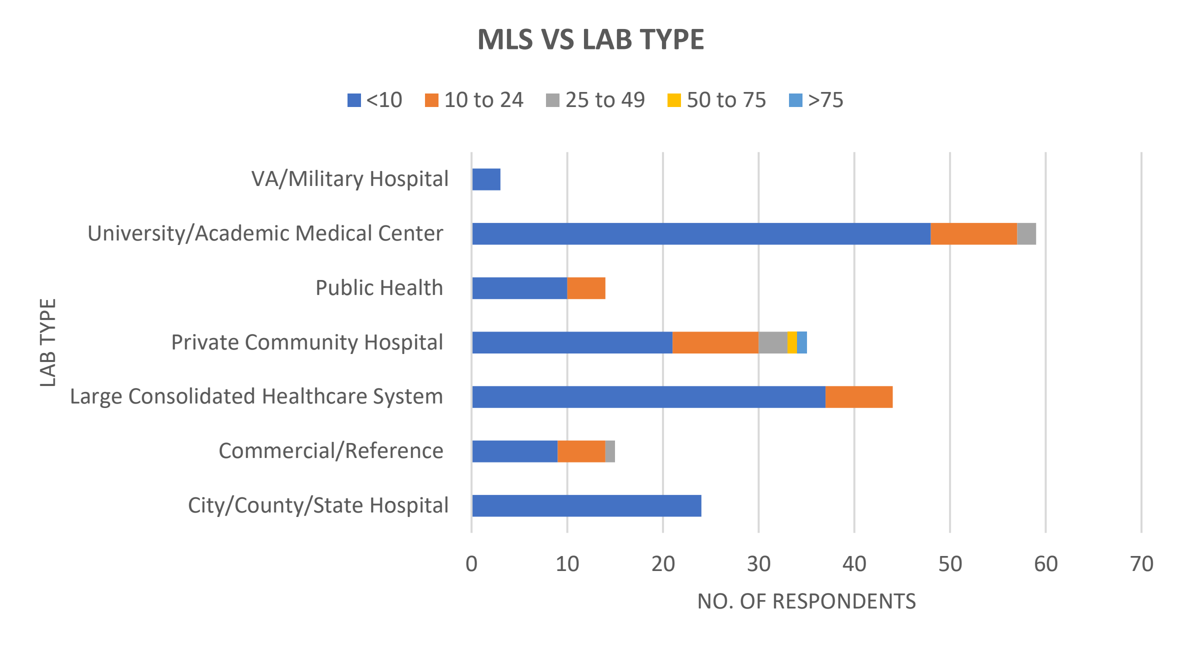

	MLS Vacancies N(% of lab group)				
Lab Type	0	1 - 2	3 - 5	6 - 10	>10
City/County/State Hospital (n=24)	7 (29.2)	16 (66.7)	1 (4.2)	0 (0)	0 (0)
Commercial/Reference (n=15)	3 (20.0)	5 (33.3)	3 (20.0)	2 (13.3)	2 (13.3)
Large Consolidated Healthcare System (n=44)	7 (15.9)	16 (36.4)	16 (36.4)	4 (9.1)	1 (2.3)
Private Community Hospital (n=35)	8 (22.9)	19 (54.3)	7 (20.0)	1 (2.9)	0 (0)
Public Health (n=14)	3 (21.4)	5 (35.7)	5 (35.7)	0 (0)	1 (7.1)
University/Academic Medical Center (n=59)	11 (18.6)	18 (30.5)	19 (32.2)	9 (15.3)	2 (3.4)
VA/Military Hospital (n=3)	0 (0)	3 (100)	0 (0)	0 (0)	0 (0)

A10. Region of the U.S versus Vacant Positions for Medical Laboratory Scientists.
Vacancies were seen in all regions of the U.S.

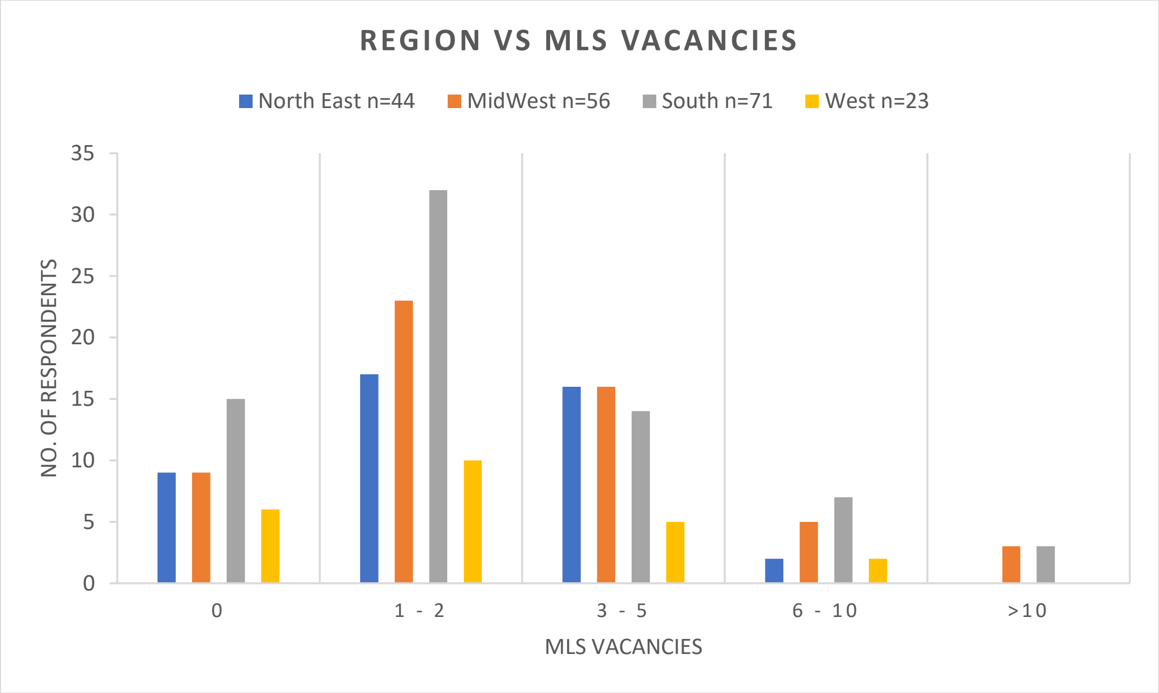

	MLS Vacancies N(% of region group)				
Region	0	1 - 2	3 – 5	6 - 10	>10
North (n=44)	9 (20.5)	17 (38.6)	16 (36.4)	2 (4.5)	0 (0)
Midwest (n=56)	9 (16.1)	23 (41.1)	16 (28.6)	5 (8.9)	3 (5.4)
South (n=71)	15 (21.1)	32 (45.1)	14 (19.7)	7 (9.9)	3 (4.2)
West (n=23)	6 (26.1)	10 (43.5)	5 (21.7)	2 (8.7)	0 (0)

North = CT, DE, MA, ME, NH, NJ, NY, PA, RI, VT
Midwest = IA, IL, IN, KS, MI, MN, MO, ND, NE, OH, SD, WI
South = AL, AR, DC , FL, GA, KY, LA, MD, MS, NC, OK, SC, TX, TN, VA, WV
West = AK, AZ, CA CO, HI, ID, MT, NM, NV, OR, UT, WA, WY

A11: Microbiology Laboratory Director versus Annual Test Volume

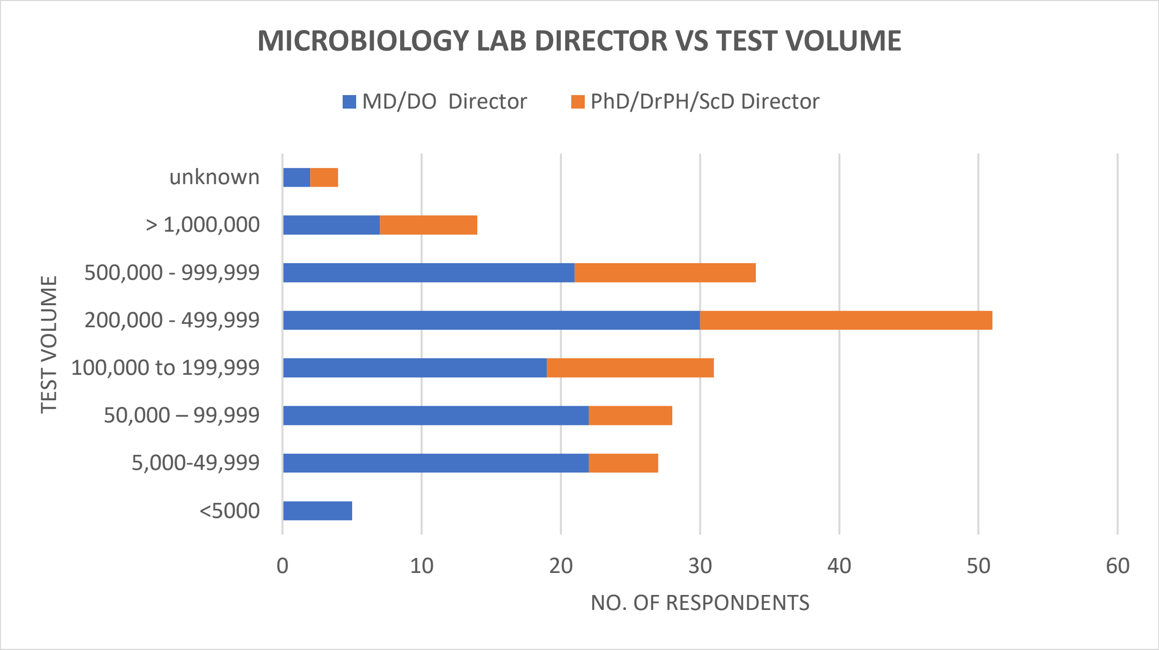

	Test Volume N(% of lab director group)							
Microbiology Lab Director	<5000	5,000-49,999	50,000 – 99,999	100,000 - 199,999	200,000 - 499,999	500,000 - 999,999	>1,000,000	unknown
MD/DO Director (n=128)	5 (3.9)	22 (17.2)	22 (17.2)	19 (14.8)	30 (23.4)	21 (16.4)	7 (5.5)	2 (1.6)
PhD/DrPH/ScD Director (n=66)	0 (0)	5 (7.6)	6 (9.1)	12 (18.2)	21 (31.8)	13 (19.7)	7 (10.6)	2 (3.0)

A12. Microbiology Laboratory Director versus Annual Test Volume

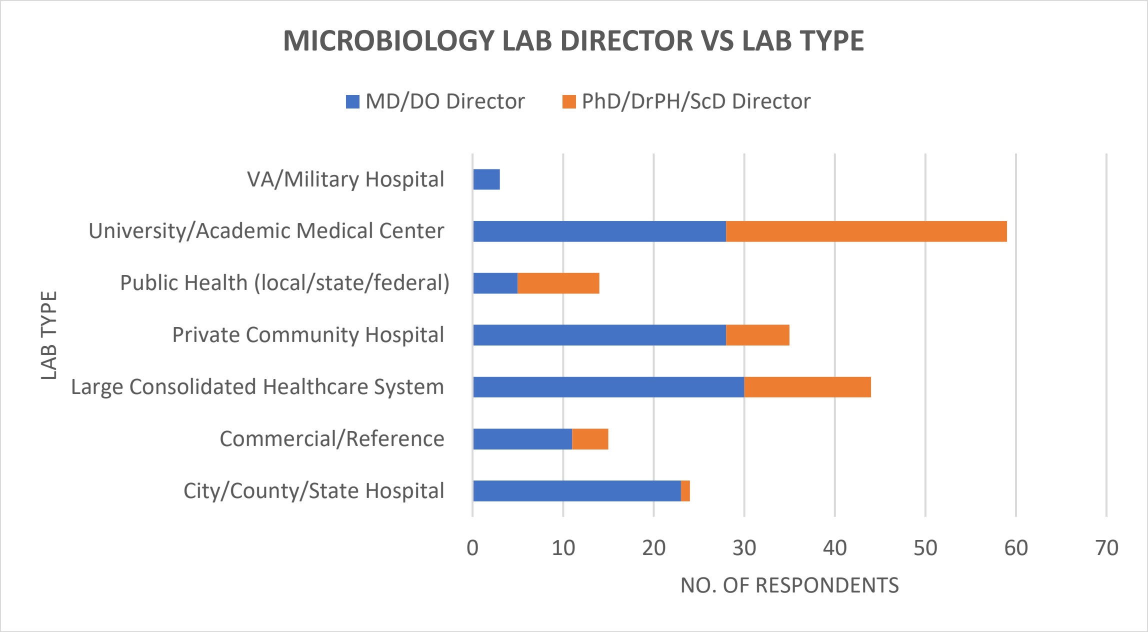

	MD/DO Director	PhD/DrPH/ScD Director
City/County/State Hospital	23 (18.0)	1 (4.2)
Commercial/Reference	11 (8.6)	4 (26.7)
Large Consolidated Healthcare System	30 (23.4)	14 (31.8)
Private Community Hospital	28 (21.9)	7 (20.0)
Public Health (local/state/federal)	5 (3.9)	9 (64.3)
University/Academic Medical Center	28 (21.9)	31 (52.5)
VA/Military Hospital	3 (2.3)	0 (0)
